# Assessment of optimized FRET substrates as universal corona- and picornavirus main protease substrates for screening assays†

**DOI:** 10.1039/d4ra06573e

**Published:** 2024-11-05

**Authors:** Conrad Fischer, Tayla J. Van Oers, Marco J. van Belkum, Tess Lamer, Aaron Romney, Pu Chen, M. Joanne Lemieux, John C. Vederas

**Affiliations:** a Department of Chemistry, University of Alberta Edmonton AB T6G 2G2 Canada john.vederas@ualberta.ca; b Department of Biochemistry, Membrane Protein Disease Research Group, University of Alberta Edmonton AB T6G 2R3 Canada

## Abstract

Coronaviral infections are an important cause of enteric and respiratory diseases in humans and animals that are generally associated with a high level of morbidity and mortality. Similarly, picornavirus infections can lead to various illnesses that severely impact human and animal health. Despite belonging to different virus families, viral replication in all of these pathogens relies on the action of a central cysteine protease called 3C/3CL or main protease (M^pro^). Due to the high functional and structural conservation of this enzyme among viral species and robustness against mutation it is considered a good target for antiviral inhibitor development. The evaluation of inhibitor potency, expressed as IC_50_, in many studies is achieved by measuring the inhibition of cleavage of a fluorogenic substrate in a Fluorescence Resonance Energy Transfer (FRET)-type assay. The FRET substrate is engineered after common recognition sequences of each viral M^pro^, resulting in different sequences and limited comparability of IC_50_ between species. Our aim was to overcome this inconsistency by identifying common recognition motives of coronavirus and picornavirus M^pro^s to develop a unique FRET substrate that can be used universally for FRET assay tests of these enzymes. We synthesized a variety of FRET substrates with common recognition sequences and compared their cleavage kinetics towards main proteases from different species to determine the optimal sequence for universal application in FRET assays.

## Abstract 2

A short FRET peptide was identified as a universal substrate for selected coronavirus M^pro^s. Kinetic parameters, buffer stability and Z'-assay suggest this substrate as an excellent target for CoV-M^pro^ HT inhibitor screening assay development.

## Graphical abstract

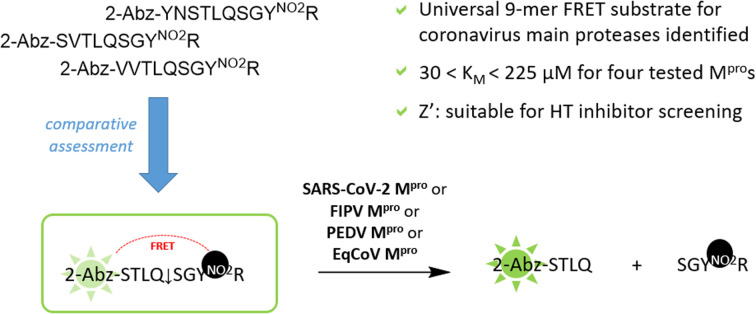

## Introduction

**Fig. 1:**
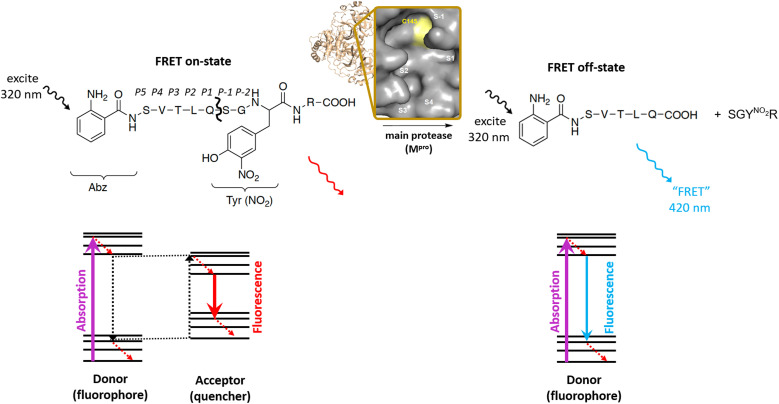
Working principle of main protease FRET substrate, exemplified for SARS-CoV-2 M^pro^. Schechter–Berger notation is highlighted for the substrate (P5–P2′) and the enzyme with the active site cavity (S1′–S4).

*Pisoniviricetes* is a class of positive-strand RNA viruses which frequently infect vertebrates. From this class, corona- and picornavirus infections can lead to severe respiratory and endemic diseases that constitute a constant and costly threat for human and animal health. Despite different pathological implications, corona- and picornaviruses share a somewhat similar biogenetic fingerprint with regard to the viral enzymes involved in the infection cycle. One key enzyme with high structural conservation and mutational robustness in these viruses is the main protease (M^pro^), also named 3C protease for picornaviruses and 3CL protease for coronaviruses, that acts as an excellent target for development of antiviral drugs.^1–3^ This main protease supports viral replication by cleavage of the viral polyprotein into non-structural and in case of picornaviruses also structural proteins. A chymotrypsin-like fold is a common structural characteristic shared by these enzymes and consists of the active site Cys residue as part of a catalytic dyad with His, in the case of coronaviruses (Fig. S1†), or as part of a triad with His and Glu (or Asp), in picornaviruses (Fig. S2†).^4^ Enzymatic characterization and inhibitor selection normally starts with *in vitro* molecular tests requiring a fluorogenic substrate, that upon cleavage by the target proteases emits a fluorescence signal. Most of these probes are designed resembling a FRET-type oligopeptide with a fluorophore unit close to one terminus and a quencher group at or near the other terminus of the peptide. In this “on-state” a portion of the emission energy of the fluorophore is transferred to and consumed by the quencher, leading to a less energetic fluorescence emission.^5^ Once the viral main protease separates the quencher from the fluorophore, shorter wavelength fluorescence emission can be detected (“off state”, Fig. 1). Corresponding to the protease of interest, numerous M^pro^ assays have been developed, using different FRET acceptor–donor combinations (Table 1), M^pro^ constructs of various length and binding affinity and assay buffer conditions.^6–9^ This unnecessarily complicates comparison of inhibitor potencies for a single target protease as well as between different corona- and picornavirus species. However, such a comparison is much needed for the development of broad-spectrum inhibitors that can universally target different viral pathogens. We thus aimed to establish a universal FRET substrate that is processed with high efficiency and optimal fluorescence characteristics by a variety of M^pro^s from *Pisoniviricetes*. We selected the M^pro^s from four coronaviruses (*i.e.* severe acute respiratory syndrome coronavirus (SARS-CoV-2), feline infectious peritonitis virus (FIPV), porcine epidemic diarrhea virus (PEDV) and equine coronavirus (EqCoV)), three picornaviruses (*i.e.* poliovirus (PV), human rhinovirus (HRV), enterovirus A71 (EV71)), and a calicivirus (*i.e.* norovirus (NV)) and cross-tested them against literature-known FRET substrates. We first summarize the chemical landscape of fluorophore–quencher pairs used in the literature for the FRET assays to identify one chemically robust pair that can be used for all herein discussed FRET substrates. This fluorophore–quencher pair is incorporated in known substrate recognition sequences of all 8 investigated main proteases and the kinetic parameters for all FRET substrates are determined. Comparison of these data suggests a universal substrate with similar cleavage kinetics for the four investigated coronavirus M^pro^s. Contrarily, among the tested FRET substrates no universally acting compound for picornaviral main proteases could be established.

**Table 1:** Characteristics of commonly used FRET fluorophores in main protease assays reported in the literature^a^

Fluorophore	Ex (nm)	Em (nm)	*R*_o_ (Förster, nm)	*Φ*_FL_ (buffered) water, pH 7–7.4	Typical quencher
AMC, 7-amino-4-methylcoumarin	351	431		0.86 (ref. 10)	—
MCA, 7-methoxycoumarin-4-acetic acid	325	420	3.7 (ref. 10)	0.72 (ref. 10)	Dnp (2,4-dinitrophenyl)
2-Abz, 2-aminobenzoic acid	320	420	3.1 (ref. 11)	0.64 (ref. 12)	Y(NO_2_) (3-nitrotyrosine)
EDANS, 5-(2-aminoethylamino)-1-naphthalenesulfonic acid	340	490	3.3 (ref. 13)	0.27 (ref. 14)	Dabcyl (4-(4-dimethylaminophenylazo)benzoyl)
FAM, 5-carboxyfluorescein	492	518	∼4.9 (ref. 15)	0.83 (ref. 16)	Dabcyl (4-(4-dimethylaminophenylazo)benzoyl)

a Abbreviations: *Φ*_FL_, fluorescence quantum yield.

## Results and discussion

### Choice of fluorophore–quencher pair

With the exception of a short fluorogenic AMC substrate that is used without a quencher group,^17^ commonly used coronavirus and picornavirus main protease FRET substrates are 8–14 amino acids in length and include a fluorogenic group and a quencher unit in proper distance.^6–9^ Once the quencher group is removed *via* proteolytic cleavage of a central peptide bond, fluorescence is emitted that is measured as the basis of the assay (Fig. 1). Noteworthy, the use of all fluorophore–quencher pair FRET substrates is limited to lower concentrations (≪100 μM) due to the inner filter effect,^18^ which is warranted in the context of most inhibitor studies. A recent study^6^ compared different literature-known FRET substrates for SARS-CoV-2 M^pro^ with additionally designed substrates based on six polyprotein cleavage sequences in a quest to find an optimized FRET substrate system suited for high-throughput screening applications (HTS). Within the selected fluorophores, that study identified a 5-carboxyfluorescein (FAM)-based FRET compound as ideal fluorogenic substrate due to a higher fluorophore brightness and green-shift driven higher accuracy. We have summarized alternative fluorophore–quencher pairs and characteristics in Table 1. Among them, for the synthesis of our FRET substrates, we selected the 2-Abz/Y(NO_2_) system as default donor–acceptor pair for its overall small molecular size, large stokes shift, high fluorescence quantum yield, and easy synthetic accessibility.

### Consensus sequence and FRET substrate length

**Fig. 2:**
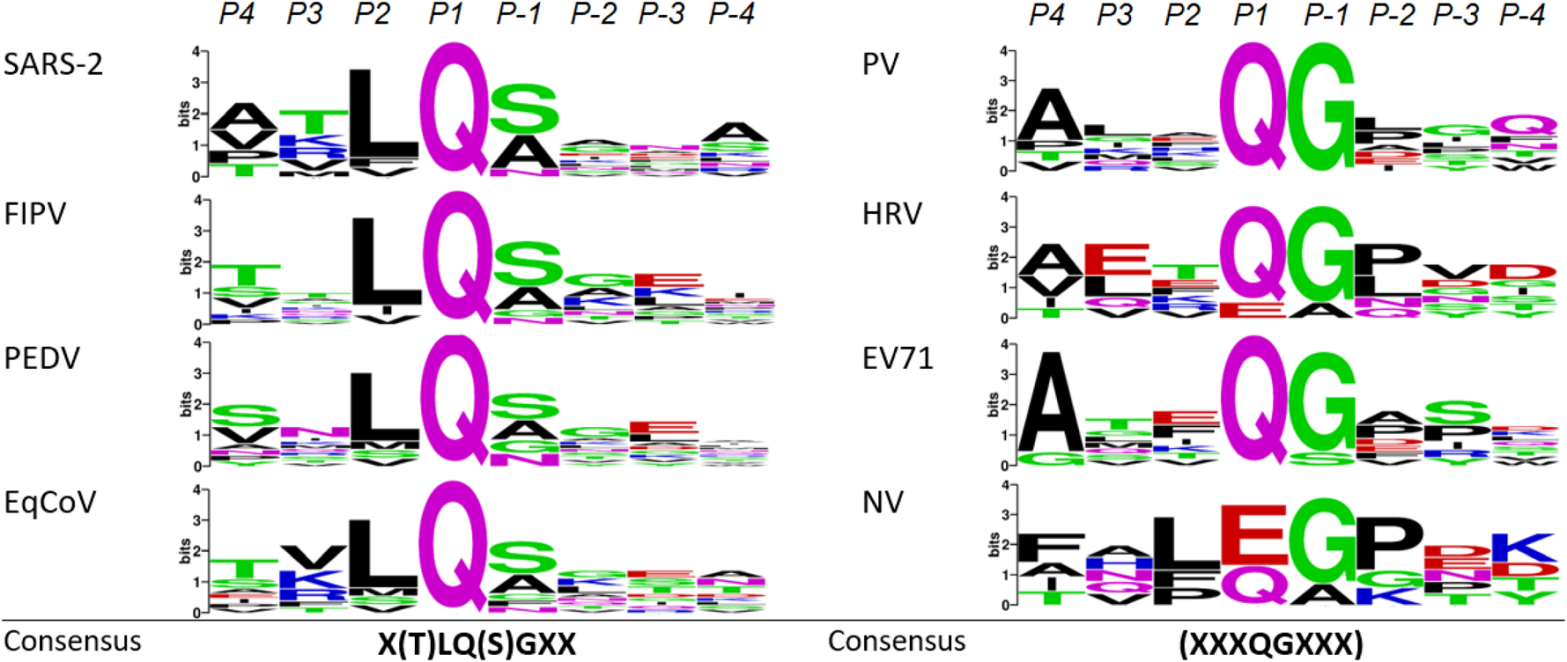
Sequence logos (WebLogo) for all investigated coronaviruses (left column), picornaviruses and norovirus (right column) cleavage sequences highlighting higher cleavage specificity in the former and intra-species variability in the latter species.

To explore the possibility of finding a FRET substrate that can be used universally across coronavirus and picornavirus main protease studies, one needs to evaluate how common the processing characteristics and recognition sequences are among the investigated proteases. This aspect shows differences between coronaviruses and picornaviruses. Coronavirus main proteases generally possess high sequence similarity and are structurally conserved among species.^19^ They cleave the translated viral polyprotein 11 times with the aforementioned preference for glutamine in P1 and a nonpolar residue, often leucine, in P2. Comparing the sequence logo of the four investigated coronavirus main proteases which displays sequence recognition patterns across the 11 cleavage sites (Fig. 2) additionally reveals a preference of serine or alanine in P-1 followed by a small, preferably neutral residue in P-2. Based on this and considering currently used coronavirus M^pro^ FRET substrates, it should be possible to extract a common sequence that is recognized by all four proteases. In fact, the FRET substrate that resembles the nsp4–nsp5 cleavage site of SARS-CoV-M^pro^ developed by Blanchard *et al.*^8^ has been successfully used in studies of the feline coronavirus version of this protease.^20^ A recent study confirms the nsp4–nsp5 site as a kinetically favoured cleavage site of SARS-CoV-2 M^pro^ and thus a good target for FRET substrate design, reflecting the self-excision site of the main protease that then catalyses further polyprotein cleavages.^21^ Structural optimization suggests a version of the original Blanchard substrate with a serine to valine substitution in P5 that is more active and results in improved fluorescence read-outs.^22^ We thus included both, Blanchard's original (Blanchard) and the modified FRET substrate (Blanchard-VV) in our studies (Table 2, Fig. S3 and S4†). For PEDV, a similar substrate has been suggested that is designed after the nsp4–nsp5 cleavage site (PEDV 1).^23^ Since the originally proposed PEDV FRET substrate (PEDV 1) shows only moderate steady state kinetics and is quite long (11 amino acids), we also included truncated and sequence optimized versions of this substrate (PEDV 2–PEDV 4) along with a negative control ((−) FRET) that does not possess the aforementioned cleavage site (Table 2 and Fig. S5–S9†). The equine protease version (EqCoV M^pro^) has yet not been characterized in the literature and therefore reflects an ideal target for establishing a suitable FRET substrate by cross-testing of the herein described compounds.

**Table 2:** Investigated FRET substrates for coronavirus M^pro^s^a^

FRET substrate	Structure	Length (amino acids)
Blanchard	2-Abz-SVTLQ↓SGY^(NO_2_)^R	10
Blanchard-VV	2-Abz-VVTLQ↓SGY^(NO_2_)^R	10
PEDV 1	2-Abz-YNSTLQ↓SGY^(NO_2_)^R	11
PEDV 2	2-Abz-NSTLQ↓SGY^(NO_2_)^R	10
PEDV 3	2-Abz-STLQ↓SGY^(NO_2_)^R	9
PEDV 4	2-Abz-STLQ↓AGY^(NO_2_)^R	9
(−) FRET (neg. control)	2-Abz-STLAAGY^(NO_2_)^R	9

a Abbreviations: 2-Abz, 2-aminobenzoic acid; Y^(NO_2_)^, 3-nitrotyrosine.

For the design of a universal FRET substrate for picornaviruses and the norovirus we followed a similar approach by first identifying polyprotein cleavage sequences for the individual M^pro^s. Unlike coronaviral M^pro^s that predominantly cleave the native polyprotein at specific positions, picornavirus main proteases expand their action to host protein and co-factor cleavage.^24^ Moreover, the number of polyprotein cleavage sites cut by either M^pro^ or the second picornaviral protease, 2A^pro^, varies greatly and suggests that the cleavage sites recognized by picornaviral M^pro^s are very species-specific.^25^

This leads to individual processing characteristics that allow more cleavage promiscuity as reflected by more variable sequence logos (Fig. 2). Generally, FRET substrates for picornavirus M^pro^s are engineered after the so-called 2C/3A junction, which again corresponds to the self-excision site of M^pro^ in these viruses. For HRV for example, it has been shown that cleavage at this site of the polyprotein happens fastest.^26^ We identified the dominant cleavage sequences in HRV^26^ PV,^27^ EV71,^28^ and NV^29–31^ and generated four FRET substrates with the 2-Abz/Y(NO_2_) pair as the basis for establishing a universal FRET substrate (Table 3 and Fig. S10–S13†).

**Table 3:** Investigated FRET substrates for picorna- and calicivirus M^pro^s^a^

FRET substrate	Structure	Length (amino acids)
HRFRET	2-Abz-ETLFQ↓GPVY^(NO_2_)^R	11
PFRET	2-Abz-EALFQ↓GPLQY^(NO_2_)^R	12
EV71FRET	2-Abz-EALFQ↓GPPKY^(NO_2_)^R	12
NVFRET	2-Abz-EFQLQ↓GKMYDY^(NO_2_)^R	13
NPFRET	2-Abz-DFHLQ↓GPY^(NO_2_)^R	10

a Abbreviations: 2-Abz, 2-aminobenzoic acid; Y^(NO_2_)^, 3-nitrotyrosine.

Additionally, we included a shorter sequence substrate (NPFRET, Table 3 and Fig. S14†) in our studies that was recently disclosed as a substrate with potential broader applicability.^7^

### Kinetic comparison

We isolated and purified the M^pro^s of SARS-CoV-2, FIPV, PEDV and EqCoV (Fig. S15–S17†) and cross-tested them against 4 selected FRET substrates, namely Blanchard,^8^Blanchard-VV,^22^PEDV 3, and PEDV 4.^23^ All proteases swiftly cleave the tested substrates as expected at the QS cleavage site, resulting in the formation of two cleavage products that can be observed by LCMS analysis (Fig. S22 and S23†). Both Blanchard substrates show *K*_M_ values ≤ 100 μM for SARS-CoV-2, FIPV, and PEDV M^pro^, but lack binding affinity for the equine version (EqCoV, Table 4). As proposed in the literature,^22^ the modified Blanchard-VV substrate demonstrates improved binding, indicated by even lower *K*_M_ values. While the shorter PEDV 4 substrate gives the lowest *K*_M_ for equine M^pro^ (165 μM), FRET peptide PEDV 3 has the overall lowest *K*_M_ values across all tested coronavirus M^pro^ targets (30–225 μM). These values are in line with, and in the case of PEDV 3, even remain under, *K*_M_ values determined for related SARS-CoV-2 M^pro^ FRET substrates.^20,22,32^ Considering substrate specificity, all investigated coronavirus M^pro^ FRET substrates show generally high turnover numbers resulting in catalytic efficiencies between 7450 and 127 600 s^−1^ M^−1^ (Table 5 and Fig. S24–S28†). Here again, the shorter PEDV 3 and PEDV 4 substrates demonstrate highest efficiencies, exceeding those observed in the literature for related SARS-CoV-2 M^pro^ substrates by 2–4 times.^20,29^ To study whether truncation of the substrate results in higher efficiency, we compared the kinetic parameters of four differently long PEDV FRET substrates, *i.e.*PEDV 1–PEDV 4 towards PEDV M^pro^. Indeed, the original PEDV FRET substrate with 11 amino acids (PEDV 1) shows the largest *K*_M_ and lowest catalytic efficiency of all four substrates (Table 6 and Fig. S27†). A general trend can be observed that efficiency increases with truncation of the substrate. Although catalytically relevant to PEDV M^pro^ cleavage,^23,33^ exchange of a P-1 serine to alanine in the truncated PEDV 4 FRET substrate results in reduced catalytic efficiency (38 600 s^−1^ M^−1^ for PEDV 4*vs.* 70 800 s^−1^ M^−1^ for PEDV 3, Table 6). Reflecting on catalytic performance, the PEDV 3 FRET substrate could be considered universally active for all investigated coronavirus M^pro^s and is forwarded as a lead compound for further assays to optimize assay conditions and warrant validity in HTS (see next chapters). None of the investigated M^pro^s recognize a control substrate that lacks the QS cleavage site ((−) FRET), highlighting the aforementioned cleavage specificity of coronavirus main proteases (compare Fig. S22 and S23†).

**Table 4:** Summary of *K*_M_ values (μM) of different FRET substrates for individual coronaviruses M^pro^s

	Blanchard	Blanchard-VV	PEDV 3	PEDV 4
SARS-CoV-2 M^pro^	82 ± 14	64 ± 10	30 ± 8	142 ± 18
FIPV M^pro^	72 ± 12	85 ± 15	33 ± 12	33 ± 11
PEDV M^pro^	100 ± 18	85 ± 10	38 ± 9	65 ± 8
EqCoV M^pro^	580 ± 92	371 ± 70	225 ± 46	165 ± 38

**Table 5:** Summary of catalytic efficiency (*k*_cat_/*K*_M_, s^−1^ M^−1^) of different FRET substrates for individual coronaviruses M^pro^s

	Blanchard	Blanchard-VV	PEDV 3	PEDV 4
SARS-CoV-2 M^pro^	33 650 ± 3200	45 600 ± 6200	79 300 ± 7400	15 500 ± 1900
FIPV M^pro^	50 800 ± 4400	38 000 ± 2950	114 000 ± 10 300	127 600 ± 9830
PEDV M^pro^	48 900 ± 3800	42 300 ± 4400	70 800 ± 6550	38 600 ± 3610
EqCoV M^pro^	7450 ± 520	12 200 ± 1100	13 800 ± 1050	24 050 ± 2050

**Table 6:** Kinetic parameters of differently long PEDV FRET substrates towards PEDV M^pro^

	PEDV 1	PEDV 2	PEDV 3	PEDV 4
*K*_M_ (μM)	192 ± 26	63 ± 11	38 ± 11	65 ± 12
*k*_cat_ (s^−1^)	3.3 ± 0.3	2.6 ± 0.2	2.7 ± 0.2	2.5 ± 0.1
*k*_cat_/*K*_M_ (s^−1^ M^−1^)	17 100 ± 1620	41 200 ± 3900	70 800 ± 6550	38 600 ± 3610

To evaluate the applicability of the PEDV 3 and the synthesized picornavirus FRET substrates in picornavirus and norovirus main protease assays, we isolated and purified the main proteases of human rhinovirus (HRV), poliovirus (PV), enterovirus A71 (EV71) and norovirus (NV) (Fig. S18–S21†). Individual FRET substrates for each viral main protease were synthesized incorporating the 2-Abz/Y(NO_2_) pair (Table 3). Together with the universal coronavirus M^pro^ lead FRET substrate PEDV 3 and a truncated substrate NPFRET (Table 3) these were cross-tested against the noroviral and picornaviral main proteases to potentially identify a universal substrate. Unfortunately, the PEDV 3 substrate is not recognized by any of the four tested proteases hinting little overlap in substrate recognition between coronavirus and picornavirus main proteases (Table 7). Steady state kinetics provide *K*_M_ values for all tested FRET substrates that are, on average, one order of magnitude bigger than determined *K*_M_s of coronaviral FRET substrates (193–3200 μM, Table 7 and Fig. S29–S32†), despite optimized sequences for each individual FRET substrate that reflect the N-terminal cleavage site of each picornavirus M^pro^. The HRFRET is only specific to rhinovirus M^pro^, and the NVFRET to enterovirus and norovirus M^pro^. Two substrates, *i.e.*PFRET and EV71FRET are somewhat more promiscuous, although with significantly higher *K*_M_ values that exceed an acceptable range for application in a universal assay.

**Table 7:** Summary of *K*_M_ values (μM) of different FRET substrates for individual picornavirus and norovirus M^pro^s^a^

	PEDV 3	HRFRET	PFRET	EV71FRET	NVFRET	NPFRET
Polio M^pro^	n.d.*	n.d.*	566 ± 182	1110 ± 240	n.d.*	n.d.*
HRV M^pro^	n.d.*	340 ± 49	193 ± 21	480 ± 80	n.d.*	n.d.*
EV71 M^pro^	n.d.*	n.d.*	3220 ± 470	1640 ± 261	1100 ± 201	n.d.*
NV M^pro^	n.d.*	n.d.*	265 ± 84	620 ± 153	613 ± 188	n.d.*

a No exact data for entries with (*) could be determined due to lack of convergence.

Contrary to the coronavirus M^pro^ FRET substrates, truncation of the picornavirus M^pro^ FRET peptides did not increase recognition as illustrated by the lack of binding of NPFRET, a truncated consensus substrate proposed in the literature.^7^ While sequence promiscuity is tolerated within each individual picornavirus M^pro^ (Fig. 2), there seems to be no consensus sequence that is universally recognized across all investigated picornavirus M^pro^s. Looking at the catalytic efficiency data (Table 8) it is also noticeable that processing of substrates that do get recognized by picornavirus M^pro^s happens on a 5 to 10 fold slower time-scale than for coronavirus species. This effect has been repeatedly observed in the literature describing picornavirus M^pro^ assays^7^ and could correlate with an extended cleavage capacity of picornavirus M^pro^ that aside from the viral polyprotein also includes processing of host proteins and co-factors. It was thus not possible to extend the idea of a universal FRET substrate to picornavirus M^pro^s.

**Table 8:** Summary of catalytic efficiency (*k*_cat_/*K*_M_, s^−1^ M^−1^) of different FRET substrates for individual picornavirus and norovirus M^pro^s^a^

	PEDV 3	HRFRET	PFRET	EV71FRET	NVFRET	NPFRET
Polio M^pro^	n.d.*	n.d.*	2500 ± 210	2700 ± 315	n.d.*	n.d.*
HRV M^pro^	n.d.*	3000 ± 285	7100 ± 890	3200 ± 405	n.d.*	n.d.*
EV71 M^pro^	n.d.*	n.d.*	890 ± 62	1300 ± 138	3400 ± 455	n.d.*
NV M^pro^	n.d.*	n.d.*	7300 ± 560	4500 ± 515	1300 ± 167	n.d.*

a No exact data for entries with (*) could be determined due to lack of convergence.

### Buffer optimization

**Fig. 3:**
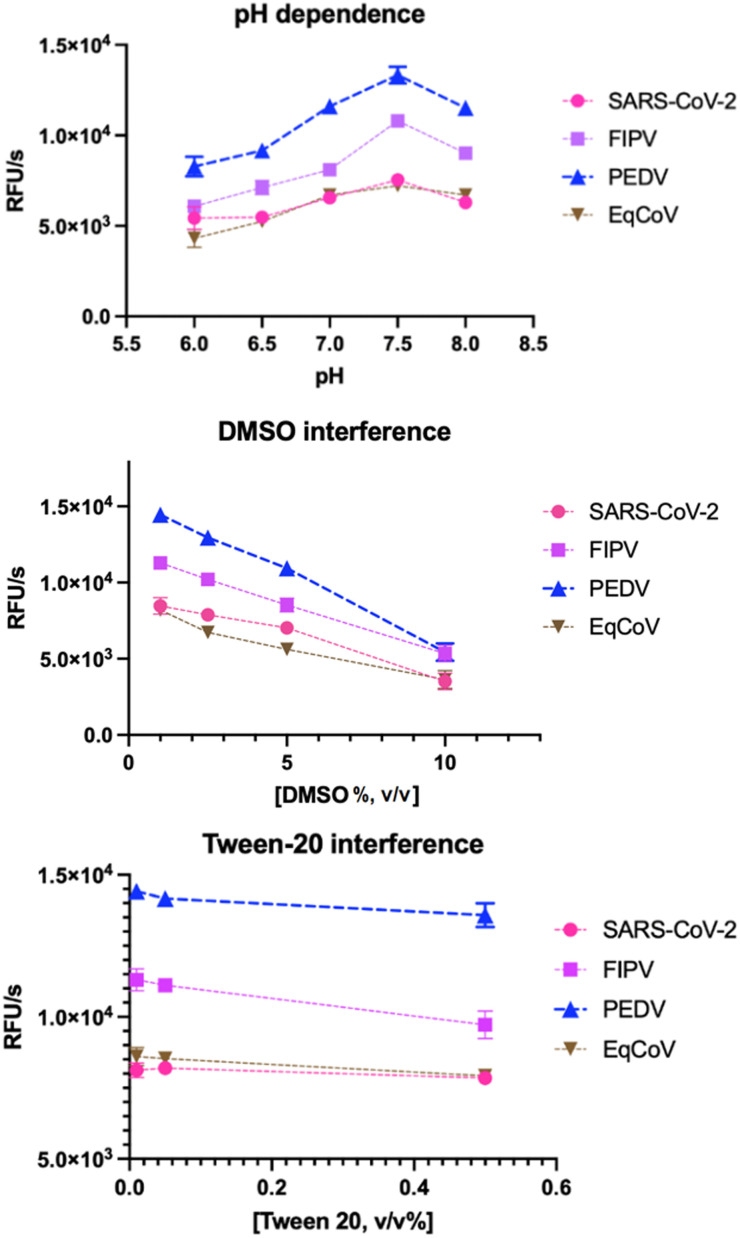
M^pro^ activity impact of common buffer conditions. Tests are done in 100 μL 20 mM Bis–Tris buffer, 1 mM DTT and 100 nM enzyme. DMSO, dimethyl sulfoxide.

To establish optimal conditions for the development of using PEDV 3 as a prospective universal coronavirus M^pro^ FRET substrate, we tested the effect of different buffer conditions on the activity of the coronaviral M^pro^s. As a standard buffer, 20 mM Bis–Tris, 1 mM dithiothreitol (DTT) with no additional salt was used. This buffer has been demonstrated as a reliable FRET assay buffer matrix in previous experiments.^6,8,20^ A pH around 7.5 was found optimal for enzymatic activity of all proteases (Fig. 3a) with SARS-CoV-2 M^pro^ and EqCoV M^pro^ being least susceptible to smaller pH changes. In accordance with previous reports^6,8^ DMSO had a negative effect, decreasing enzyme activity by 42–60% for the various proteases at 10% DMSO (Fig. 3b). It is thus recommended to keep the final DMSO concentration in the assay below 1% for maximal readout. Detergents like Tween-20 are frequently used to increase stability and solubility of the enzymes^34,35^ and do not significantly reduce catalytic activity within the tested concentration range (0.02–0.5% (v/v)) (Fig. 3c). We did not test the effect of higher salt (NaCl) and glycerol concentrations, since previous studies have already elucidated the strong inhibitory effect of these additives.^6^

### *Z*′ assay

To characterize the quality of assay data and suitability of PEDV 3 as a universal FRET substrate for high-throughput screening (HTS) we determined the *Z*′ factor of PEDV 3 for each of the four coronavirus M^pro^s under investigation.^36^ As a validation of assay quality, the *Z*′ factor includes the signal dynamic range (signal difference between positive and negative control) to reliably distinguish active from inactive compounds in inhibitor screening and the standard deviation of signals from positive and negative controls as measurement of prediction confidence. Baicalein, a non-covalent inhibitor of SARS-CoV-2,^37^ is used as a positive (+) control, and DMSO as a negative (−) control. All calculated *Z*′ factors are above 0.5 indicating high reproducibility, robustness, and reliability of the assay (Table 9 and Fig. S33†). PEDV 3 performs best in assays with SARS-CoV-2 M^pro^ (avg. *Z*′ = 0.65) and PEDV M^pro^ (avg. *Z*′ = 0.65). These values suggest PEDV 3 as a robust M^pro^ FRET substrate suitable for high-throughput screening applications.

**Table 9:** Assay quality statistics for PEDV 3 FRET substrate towards various coronavirus M^pro^s^a^

Enzyme	Replicate	Signal mean (RFU s^−1^)		SDV (RFU s^−1^)		SDR (RFU s^−1^)	*Z*′
		(+) control	(−) control	(+) control	(−) control		
SARS-CoV-2 M^pro^	1	54	6524	96	738	6470	0.613
	2	43	7116	118	622	7073	0.686
FIPV M^pro^	1	78	11 479	111	1467	11 401	0.585
	2	84	11 751	86	1648	11 667	0.554
PEDV M^pro^	1	335	14 181	496	988	13 845	0.679
	2	159	13 626	341	1370	13 467	0.619
EqCoV M^pro^	1	94	7612	70	1064	7517	0.547
	2	80	7233	74	896	7152	0.593

a Abbreviations: SDV, standard deviation; SDR, signal dynamic range.

## Conclusions

The development of main protease (M^pro^) inhibitors against coronavirus and picornavirus pathogens is of paramount interest to combat associated life-threatening human and animal diseases and depends on the reliable interpretation of results from drug screening assays. As such, M^pro^ FRET assays are frequently used for their accessibility, theoretical accuracy and quick turnaround times, however, comparability and interpretation of data between different studies of the same M^pro^ and across species is difficult due to different employed substrates, specific for each main protease. Based on FRET sequence comparison of four selected coronavirus M^pro^s, *i.e.* SARS-CoV-2, FIPV, PEDV and EqCoV we were able to design a consensus FRET substrate, *i.e.*PEDV 3, that is nearly equally recognized by all four proteases and thus might be employed/tried as universal FRET substrate across a broad spectrum of coronaviral M^pro^ targets. Optimized assay conditions and data quality validation suggest suitability for HTS applications. A similar approach across three selected picornavirus M^pro^s did not lead to a reliable universal FRET substrate for picornavirus M^pro^ assays highlighting different processing characteristics of coronaviral and picornaviral main proteases.

## Experimental

### Synthesis and purification of FRET substrates

Amino acid sequences of employed FRET substrates are disclosed in Tables 2 and 3 and were synthesized by solid phase peptide synthesis (SPPS). All commercially available reagents and protected amino acids were purchased and used without further purification unless otherwise noted. All the solvents used for reactions were used without further purification unless otherwise noted. Dry solvents refer to solvents freshly distilled over appropriate drying reagents prior to use. For each FRET substrate the first amino acid was loaded as follows: 2-chlorotrityl chloride resin was transferred to a SPPS vessel and washed with dry CH_2_Cl_2_ (2 × 10 mL) and then dry DMF (2 × 10 mL) for one min each, and then bubbled under Ar in dry DMF (10 mL) for 10 min. The desired Fmoc-protected amino acid (1.0 equiv., based on desired resin loading) and DIPEA (5.0 equiv.) were suspended in 10 mL of a 50/50 mixture of dry CH_2_Cl_2_/DMF. This solution was bubbled under Ar for 2.5 h to load the desired amino acid onto the solid support, continually topping up the CH_2_Cl_2_ to maintain an approximately 10 mL volume. To end cap any remaining trityl groups, dry MeOH was added to the vessel (0.8 mL per gram of resin) and bubbled under Ar for 15 minutes. After draining, the resin was washed with dry DMF (3 × 10 mL), dry CH_2_Cl_2_ (3 × 10 mL), and again DMF (3 × 10 mL). The resin was elongated by coupling 3 equiv. of Fmoc-protected amino acid, 3 equiv. of PyBOP (benzotriazol-1-yloxytripyrrolidinophosphonium hexafluorophosphate), 3 equiv. of HOBt (hydroxybenzotriazole) and 9 equiv. of DIPEA (*N*,*N*-diisopropyl ethylamine) in DMF for 1 hour. Fmoc residues were deprotected using a 20% solution of piperidine in DMF (3 × 7 min). The N-terminal 2-Abz building block was attached as Boc-protected amino acid using the same conditions as for Fmoc-protected amino acid couplings. To cleave the mature peptide, resin-bound analogue was suspended in 95/2.5/2.5 TFA/TIPS/H_2_O with shaking for 2–3 h. The resin was removed *via* filtration through glass wool, rinsed with TFA, and the solution concentrated *in vacuo*. Cold diethyl ether (2 × 5 mL) was added to triturate the crude residue. The diethyl ether was decanted and briefly centrifuged for 3 minutes at 13 000 rpm to pellet any residual peptide. The ether was removed, and the peptide pellet was then dried thoroughly by centrifugation in a vacuum centrifuge for 5 minutes. The pellet and triturated crude residue were pooled together and dissolved in 0.1% aqueous TFA. FRET peptides were purified using a Vydac Si C18 RP-HPLC semi-preparative column (300 Å, 5 μM, 10 × 250 mm) with aqueous 0.1% TFA (solvent A) and 0.1% TFA in acetonitrile (solvent B) as eluents. The analytical purification method used was: 0–3 min 10% B, 3–4.5 min 10–25% B, 4.5–14.5 min 25–40% B, 14.5–17 min 40–90% B, 17–19.5 min 95% B, 19.5–20.5 min 95–10% B, 20.5–30 min 10% B. The HPLC fractions were pooled and lyophilized to produce the peptides as a yellow powder. All peptides were analysed using LC and HRMS (ESI). Samples were run on an Agilent Technologies 6130 LCMS using a Core–Shell C8-column (1.7 μm, 100 A, Phenomenex Kintex). A solvent gradient (A: H_2_O with 0.1% TFA, B: ACN with 0.1% TFA) was employed as follows: 0–5 min, 2–100% B; 5–8 min, 100% B to elute each substrate as single peak (Fig. S5–S14†).

### Cloning, expression and purification of M^pro^ proteins

The cloning of the M^pro^ genes of SARS-CoV-2 and FIPV into the pET SUMO expression vector (Invitrogen), and the expression of these M^pro^ enzymes as fusion proteins with an N-terminal SUMO (small ubiquitin-like modifier) domain has been described before.^20,38^ The genes encoding the M^pro^ of PEDV (Genbank: QAR17955.1), EqCoV (GenBank: UVD39584.1), PV (Genbank: NP_740476.2), HRV (Genbank: NP_740524.1), EV71 (Genbank: AB204853.1) and NV (Genbank: NP_786949.1) were obtained from Genscript and codon optimized for expression in *Escherichia coli*. The genes were cloned into pET SUMO or pET28 SUMO vectors in such a way that the M^pro^ protein is in frame with the His-tagged SUMO protein. The resulting plasmids were transformed into *E. coli* BL21(DE3), induced by 0.5 mM isopropyl β-d-1-thiogalactopyranoside and the fusion proteins were expressed at 32 °C for 5 h. Cells were harvested by centrifugation (4000*g* for 10 min at 4 °C), resuspended in lysis buffer (20 mM Tris–HCl pH 7.8, 150 mM NaCl) and lysed by sonication. Cell debris were spun down by centrifugation (27 000*g* for 20 min at 4 °C) and after addition of 5 mM imidazole, the supernatants were loaded onto a Ni–NTA resin column (Qiagen). The resin columns were washed with 10 column volumes of lysis buffer containing 20 mM imidazole and the fusion proteins were eluted with 3 column volumes of lysis buffer containing 300 mM imidazole and 1 mM dithiothreitol (DTT). The protein samples were dialyzed against lysis buffer containing 1 mM DTT, 1 mM EDTA, and 0.002% Tween-20 for 3 h at 4 °C. After dialysis, the protein samples were concentrated using AMICON Ultra-15 filters (Millipore) with a MWCO of 10 kDa and digested with His-tagged SUMO protease (McLab) for 2 h at 4 °C to remove the SUMO tag from the fusion proteins. The protein mixtures were then loaded onto a Ni–NTA resin column and the M^pro^ proteins were obtained in the flow-through. The flow-through was further purified using size exclusion chromatography (Sephadex G-15, GE Healthcare), with buffer containing 20 mM Tris–HCl pH 7.8, 150 mM NaCl, 1 mM DTT, 1 mM EDTA, and 0.002% Tween-20. The fractions containing each M^pro^ were pooled and concentrated using an Amicon Ultra-15 filter with a MWCO of 10 kDa.

### General procedure for enzymatic assays

All assays were analysed with a Spectramax i3x microplate reader controlled by Softmax Pro software (Version 6.5.1, Molecular Devices). Readings were taken in black 96 well flat bottom polypropylene microplates (Corning) under specific time regimes (see below) at 37 °C in assay buffer (20 mM Bis–Tris, pH 7.6, 1 mM DTT, 0.02% Tween-20). Excitation and emission wavelength specific for 2-Abz were set to 320 nm (bandwidth 9 nm) and 420 nm (bandwidth 15 nm), respectively. Initial rates were fit to the linear portion of the reaction progress curve, accounting for less than 10% substrate hydrolysis. Fluorescence units were converted to concentration using a standard curve generated using a 2-Abz standard in 20 mM Bis–Tris (pH 7.6).

### Steady state enzyme kinetics

Michaelis–Menten kinetics were measured in 20 mM Bis–Tris buffer (pH 7.6), containing 1 mM DTT and 0.02% Tween-20 in a total volume of 140 μL per well. For coronavirus M^pro^ assays, 100 nM enzyme was used with FRET substrate concentrations ranging from 5 to 700 μM. For picornavirus M^pro^ assays, 500 nM enzyme was used with FRET substrate concentrations from 6 to 850 μM. Reactions were initiated by addition of enzyme and fluorescence read every 15 seconds for 10 minutes for coronavirus M^pro^s and every minute for 60 minutes for picornavirus M^pro^s using above settings. After correcting values for photo-bleaching and inner filter effect progress curves in RFU s^−1^ were converted into μM s^−1^ with the help of a calibration curve constructed with 2-Abz. Initial velocities were calculated from the linear curve part (first 90 seconds for coronavirus M^pro^s, first 300 seconds for picornavirus M^pro^s) and plotted against PEDV 3 concentrations to obtain values of *K*_M_ and *v*_max_ using the non-linear, least squares regression analysis in Graphpad Prism 9 software. To calculate *k*_cat_, *v*_max_ was divided by the molar concentration of enzyme used in each assay (as specified above). With these values of *k*_cat_ and *K*_M_, the value of *k*_cat_/*K*_M_ was subsequently calculated assuming a fixed amount of active enzyme used in the experiment.

### Assay quality assessment

The *Z*′-factor was assessed by measuring the M^pro^ activity (RFU s^−1^) of all four coronaviral main proteases towards the PEDV 3 FRET substrate for 16 positive and 16 negative controls and repeated in duplicate, reading for 6 minutes every 15 seconds. Baicalein (CAS number: 491-67-8; Sigma-Aldrich), a noncovalent inhibitor of SARS-CoV-2 M^pro^, was used as a positive control; the negative control contained DMSO. The reaction contained 100 μL of 10 μM PEDV 3 FRET substrate, 100 nM M^pro^ enzyme, and either 50 μM baicalein or DMSO as the positive and negative controls, respectively. For each assay, the mean and standard deviation of the initial rate (first 90 seconds) for positive and negative controls were calculated. The signal dynamic range was calculated according to the following, where *μ*_n_ and *μ*_p_ are the mean of the negative and positive controls, respectively.Signal dynamic range = *μ*_n_ − *μ*_p_

The *Z*′-factor was calculated according to Zhang *et al.*^36^ where *σ*_n_ and *σ*_p_ are the standard deviation of the positive and negative controls, respectively.
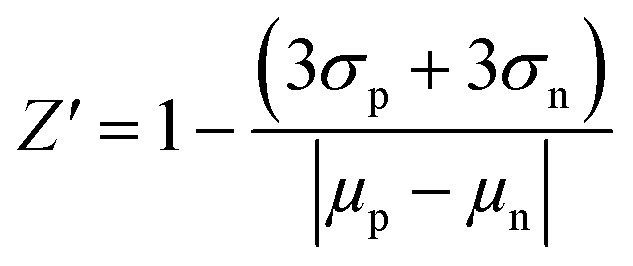


## Data availability

All data are contained within the article and in the ESI.†

## Author contributions

CF, JCV, and MJvB conceptualization; CF, TJVO, MJvB and JCV methodology; CF, TJVO, AR, and TL synthesis of FRET substrates; MJvB and MJL synthesis of M^pro^ enzymes; PC vector design; CF and TJVO investigation, analysis, and validation; CF writing – original draft; CF, MJvB, TJVO, TL, JCV and MJL writing – review & editing.

## Conflicts of interest

There are no conflicts to declare.

## Supplementary Material

RA-014-D4RA06573E-s001
