# Supplementary material for: Assessment of optimized FRET substrates as universal corona- and picornavirus main protease substrates for screening assays
Source: RSC Adv. 2024 Nov 5;14(48):35438–46. doi: 10.1039/d4ra06573e (PMC11537199; doi:10.1039/d4ra06573e)
Supplement: RA-014-D4RA06573E-s001 [file RA-014-D4RA06573E-s001.pdf]

Supplementary Information (SI) for RSC Advances.
This journal is © The Royal Society of Chemistry 2024

Supplemental information

Assessment of optimized FRET substrates as universal corona- and picornavirus main protease substrates for screening assays

Conrad Fischer,[a] Tayla J. Van Oers,[a] Marco J. van Belkum,[a] Tess Lamer,[a] Aaron Romney,[a] Pu Chen,[b] M. Joanne Lemieux[b] and John C. Vederas[a,*]

[a] Department of Chemistry, University of Alberta, Edmonton AB, T6G 2G2, Canada

[b] Department of Biochemistry, Membrane Protein Disease Research Group, University of Alberta, Edmonton AB, T6G 2R3, Canada

* correspondence author email: john.vederas@ualberta.ca

Sequence comparison of M^{pro}s from investigated coronaviruses
Figure S1. Sequence alignment of coronavirus main proteases under investigation……………..……..2
Sequence comparison of M^{pro}s from investigated picornaviruses
Figure S2. Sequence alignment of picornavirus main proteases under investigation……………..…….3
Chromatographic and mass spectroscopic data and purity of synthesized FRET substrates
Figure S3. LCMS data for **Blanchard** substrate…………………………………………………………..…4
Figure S4. LCMS data for **Blanchard-VV** substrate……………………………………………………..…4
Figure S5. LCMS data for **PEDV 1** substrate………………………………………………………..…….5
Figure S6. LCMS data for **PEDV 2** substrate………………………………………………………..…….5
Figure S7. LCMS data for **PEDV 3** substrate………………………………………………………..…….6
Figure S8. LCMS data for **PEDV 4** substrate………………………………………………………..…….6
Figure S9. LCMS data for **(-) FRET** substrate (neg. control)………………………..……………….…..7
Figure S10. LCMS data for **HRVFRET** substrate……………………………………………………..…..7
Figure S11. LCMS data for **PVFRET** substrate…………………………………………………..………8
Figure S12. LCMS data for **EV71FRET** substrate……………………………………………..…….…8
Figure S13. LCMS data for **NVFRET** substrate…………………………………………………..……….9
Figure S14. LCMS data for **NPFRET** substrate…………………………………………………..……..9
Gel filtration and LCMS data of purified tag-free main proteases
Figure S15. Chromatographic and ESI mass-spectrometric characterization of FIPV-M^{pro}……………10
Figure S16. Chromatographic and ESI mass-spectrometric characterization of PEDV-M^{pro}…………..11
Figure S17. Chromatographic and ESI mass-spectrometric characterization of EqCoV-M^{pro}…………12
Figure S18. Chromatographic and ESI mass-spectrometric characterization of HRV-M^{pro}……………..13
Figure S19. Chromatographic and ESI mass-spectrometric characterization of PV-M^{pro}………………14
Figure S20. Chromatographic and ESI mass-spectrometric characterization of EV71-M^{pro}……………15
Figure S21. Chromatographic and ESI mass-spectrometric characterization of NV-M^{pro}………………16
Kinetic data of FRET substrates for coronavirus M^{pro}s
Figure S22. Reaction progression curves of various Mpros with selected FRET substrates…………..17
Figure S23. LCMS data of SARS-CoV-2 M^{pro} cleavage products with selected FRET substrates…….18
Figure S24. Michaelis-Menten plot of various coronavirus FRETs with SARS-CoV-2 M^{pro} ……………19
Figure S25. Michaelis-Menten plot of various coronavirus FRETs with FIPV M^{pro} ……………………..…19
Figure S26. Michaelis-Menten plot of various coronavirus FRETs with PEDV M^{pro} ………………..……20
Figure S27. Michaelis-Menten plot of various PEDV FRETs with PEDV M^{pro} ……………………..…….20
Figure S28. Michaelis-Menten plot of various coronavirus FRETs with EqCoV M^{pro} ……………..……..21
Kinetic data of FRET substrates for picornavirus M^{pro}s
Figure S29. Michaelis-Menten plot of various picornavirus FRETs with HRV M^{pro} ………………..…….21
Figure S30. Michaelis-Menten plot of various coronavirus FRETs with PV M^{pro} ………………….…….22
Figure S31. Michaelis-Menten plot of various coronavirus FRETs with EV71 M^{pro} ……………..……….22
Figure S32. Michaelis-Menten plot of various coronavirus FRETs with NV M^{pro} ……………………..…….23
Z' assay for PEDV3 for various coronavirus M^{pro}s
Figure S33. Z' assay quality statistics for different coronaviral M^{pro}s…………………………….…….24

```
FIPV            SGLRKMAQPSGVVEPCIVRVAYGNNVLNGLWLGDEVICPRHVIASDTS-RVINYENELSS  59
PEDV            AGLRKMAQPSGVVEKCIVRVCYGNMALNGLWLGDTVMCPRHVIASSTT-STIDYDYALSV  59
SARS-CoV-2      SGFRKMAFPSGKVEGCMVQVTCGTTTLNGLWLDDVVYCPRHVICTSEDMLNPNYEDLLIR  60
EqCoV           SGIVKMVNPTSKVEPCIVSVTYGNMTLNGLWLDDKVYCPRHVICSASDMTNPDYTNLLCR  60
                :*: **. *:. ** *:* *  *. .******.* * ******.:       :*   *

FIPV            VRLHNFSIAKNNVFLGVVSAKYKGVNLVLKVNQVNPNTPEHKFKSVRPGESFNILACYEG  119
PEDV            LRLHNFSISSGNVFLGVVGVTMRGALLQIKVNQNNVHTPKYTYRTVRPGESFNILACYDG  119
SARS-CoV-2      KSNHNFLVQAGNVQLRVIGHSMQNCVLKLKVDTANPKTPKYKFVRIQPGQTFSVLACYNG  120
EqCoV           VTSSDFTVLFDRLSLTVMSYQMQGCMLVLTVTLQNSRTPKYTFGVVKPGETFTVLAAYNG  120
                    :* :  ..: * *:.   :.  * :.*   * .**::.:  ::**::*.:**.*:*

FIPV            CPGSVYGVNMRSQGTIKGSFIAGTCGSVGYVLENGTLYFVYMHHLELGNGSHVGSNLEGE  179
PEDV            AAAGVYGVNMRSNYTIRGSFINGACGSPGYNINNGTVEFCYLHQLELGSGCHVGSDLDGV  179
SARS-CoV-2      SPSGVYQCAMRPNFTIKGSFLNGSCGSVGFNIDYDCVSFCYMHHMELPTGVHAGTDLEGN  180
EqCoV           KPQGAFHVTMRSSYTIKGSFLCGSCGSVGYVLMGDCVKFVYMHQLELSTGCHTGTDFNGD  180
                   ..:   ** . **:***: *:*** *: :  . : * *:*::** .* *.*::::*

FIPV            MYGGYEDQPSMQLEGTNVMSSDNVVAFLYAALINGERWFVTNTSMTLESYNSWAKTNSFT  239
PEDV            MYGGYEDQPTLQVEGASSLFTENVLAFLYAALINGSTWWLSSSRIAVDRFNEWAVHNGMT  239
SARS-CoV-2      FYGPFVDRQTAQAAGTDTTITVNVLAWLYAAVINGDRWFLNRFTTTLNDFNLVAMKYNYE  240
EqCoV           FYGPYKDAQVVQLPVQDYIQSVNFVAWLYAAILNNCNWFVQSDKCSVEDFNVWALSNGFS  240
                :** : *    *    .   : *.:*:****::*.  *::     ::: :*  *   .

FIPV            EIVST--DAFNMLAAKTGYSVEKLLECI-VRLNKGFGGRTILSYGSLCDEFTPIEVIRQM  296
PEDV            TVVNT--DCFSILAAKTGVDVQRLLASI-QSLHKNFGGKQILGYTSLTDEFTTGEVIRQM  296
SARS-CoV-2      PLTQDHVDILGPLSAQTGIAVLDMCASLKELLQNGMNGRTILGSALLEDEFTPFDVVRQC  300
EqCoV           QVKSDLV--IDALASMTGVSLETLLAAI-KRLKNGFQGRQIMGSCSFEDELTPSDVYQQL  297
                 : .     :. *:: **  :  :  .:   *::.: *: *:.   : **:*  :* :*

FIPV            YGVNLQ  302
PEDV            YGVNLQ  302
SARS-CoV-2      SGVTFQ  306
EqCoV           AGIKLQ  303
                 *:.:*
```

Figure S1. Sequence alignment of coronavirus main proteases under investigation. The catalytic dyad (His41, Cys145) is highlighted in green, conserved sub-pocket residues (S1', S1 and S2) important for substrate recognition are highlighted in light grey.

```
NV        APPTLWSRVTKFGSGWGFWVS----------------------PTVFITTTHVVPTGVKE  38
HRV       ------------GPNTEFALSLLRKNIMTITTSKGEFTGLGIHDRVCVIPTHAQPGDDVL  48
PV        ------------GPGFDYAVAMAKRNIVTATTSKGEFTMLGVHDNVAILPTHASPGESIV  48
EV71      ------------GPSLDFALSLLRRNIRQAQTDQGHFTMLGVRDRLAILPRHSQPGKTIW  48
                      * .  : ::                        : :   *  *

NV        FFGE------------------PLSSIAIHQAGEFTQFRFSKKMRP-DLTGMVLEEGCPE  79
HRV       VNGQKIRVKDKYKLVDPENINLELTVLTLDRNEK---FRDIRGFISEDLE----------  95
PV        IDGKEVEILDAKALEDQAGTNLEITIITLKRNEK---FRDIRPHIPTQIT----------  95
EV71      VEHKLINVLDAVELVDEQGVNLELTLVTLDTNEK---FRDITKFIPEVIT----------  95
          .  :                   :: ::::.   :   **         :

NV        GTVCSVLIKRDSGELLPLAVRMGAIASMRIQGRLVHGQSGMLLTGA---NAKGMDLGTIP  136
HRV       G-VDATLVVH-SNNFTNTILEVGPVTM---AGL-------INLSSTPTNRMIRYDYATKT  143
PV        ETNDGVLIVN-TSKYPNMYVPVGAVTE---QGY-------LNLGGRQTARTLMYNFPTRA  144
EV71      GASDATLVIN-TEHMPSMFVPVGDVVQ---YGF-------LNLSGKPTHRTMMYNFPTKA  144
              ..*: . : .     : :* :.     *        : * .    .    :  *

NV        GDCGAPYVHKRGNDWVVCGVHAAATKSGNTVVCAVQAGEGETALE------------     181
HRV       GQCGGVLCATG----KIFGIHV--------------GGNGRQGFSAQLKKQYFVEKQ     182
PV        GQCGGVITCTG----KVIGMHV--------------GGNGSHGFAAALKRSYFTQSQ     183
EV71      GQCGGVVTSVG----KIIGIHI--------------GGNGRQGFCAGLKRSYFASEQ     183
          *:**.           : *:*               .*:*  .:
```

Figure S2. Sequence alignment of picornavirus and norovirus main proteases under investigation. The catalytic triad (His41, Cys147) is highlighted in green, conserved sub-pocket residues (S1’, S1 and S2) important for substrate recognition are highlighted in light grey.

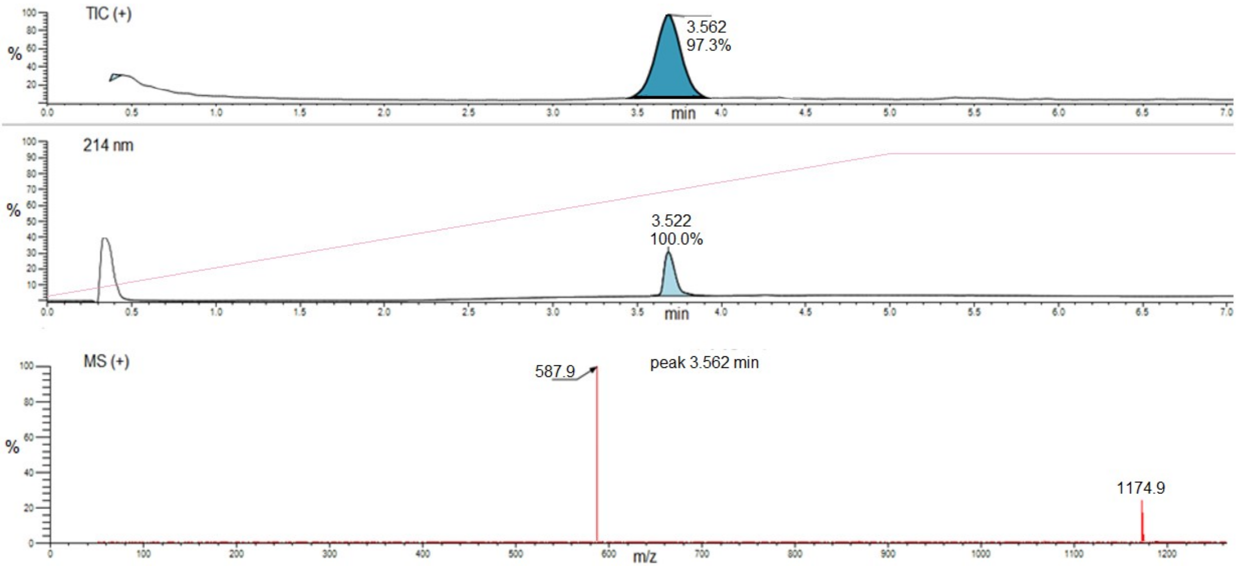

Figure S3. Total ion count chromatogram (TIC+) and mass spectral data for compound **Blanchard** after cleavage of the assembled peptide from SPPS resin and HPLC purification. The solvent gradient (%B = acetonitrile) is highlighted in pink in the DAD chromatogram at 214nm.

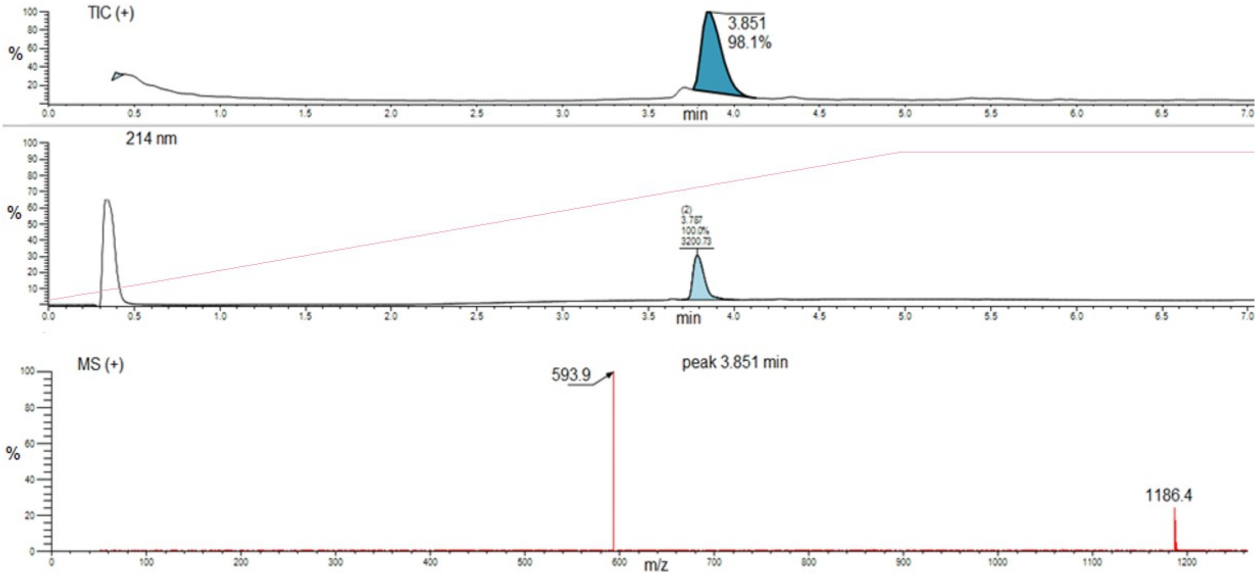

Figure S4. Total ion count chromatogram (TIC+) and mass spectral data for compound **Blanchard-VV** after cleavage of the assembled peptide from SPPS resin and HPLC purification. The solvent gradient (%B = acetonitrile) is highlighted in pink in the DAD chromatogram at 214nm.

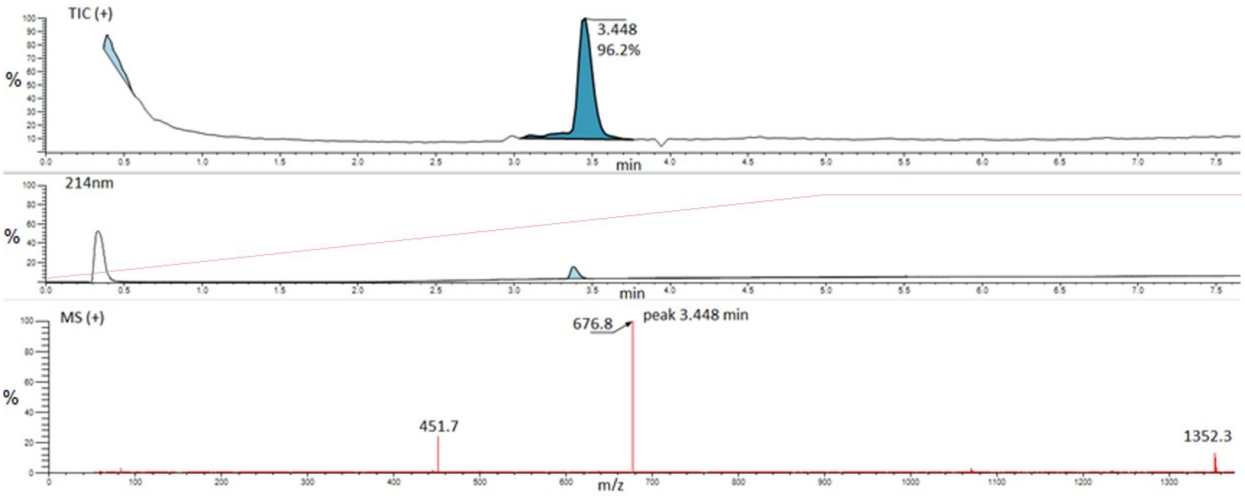

Figure S5. Total ion count chromatogram (TIC+) and mass spectral data for compound **PEDV 1** after cleavage of the assembled peptide from SPPS resin and HPLC purification. The solvent gradient (%B = acetonitrile) is highlighted in pink in the DAD chromatogram at 214nm.

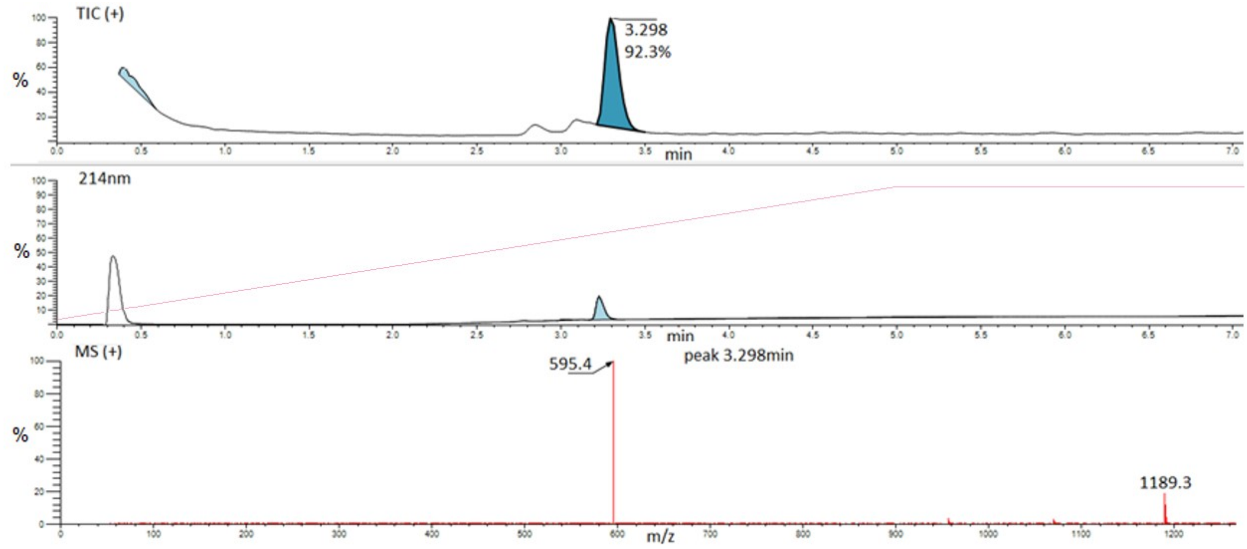

Figure S6. Total ion count chromatogram (TIC+) and mass spectral data for compound **PEDV 2** after cleavage of the assembled peptide from SPPS resin and HPLC purification. The solvent gradient (%B = acetonitrile) is highlighted in pink in the DAD chromatogram at 214nm.

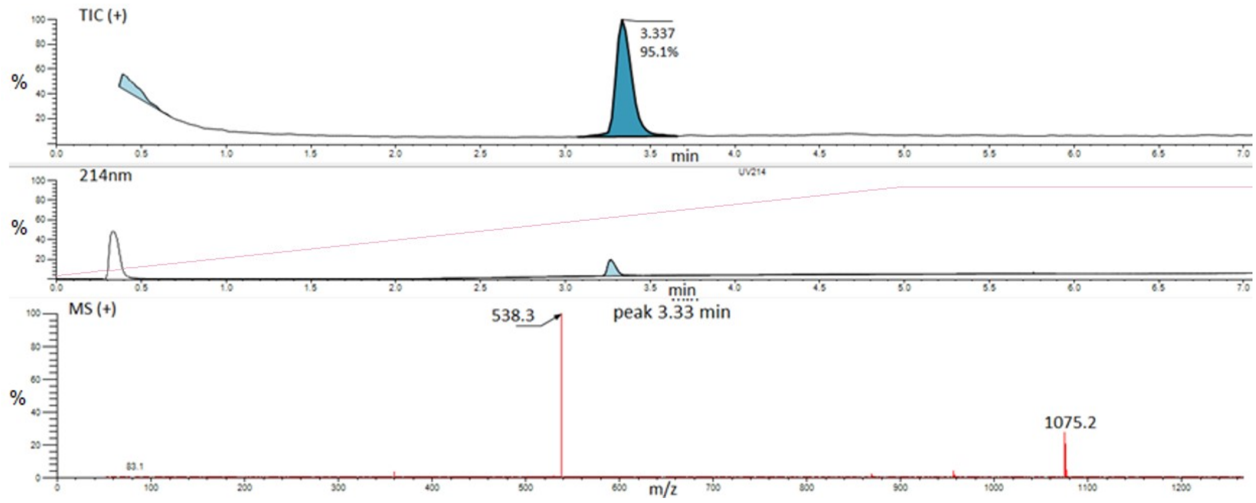

Figure S7. Total ion count chromatogram (TIC+) and mass spectral data for compound **PEDV 3** after cleavage of the assembled peptide from SPPS resin and HPLC purification. The solvent gradient (%B = acetonitrile) is highlighted in pink in the DAD chromatogram at 214nm.

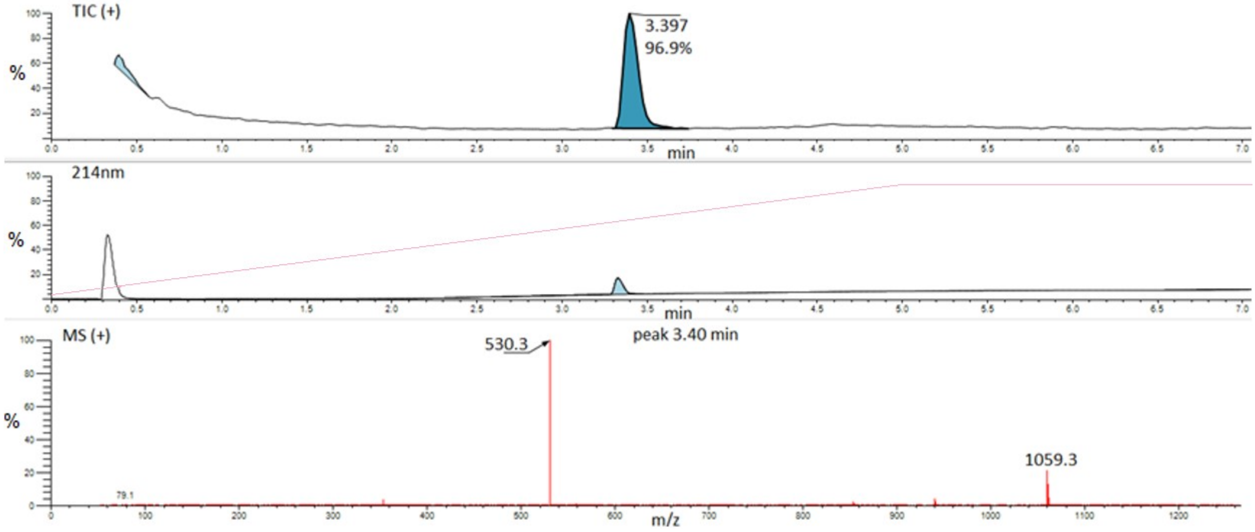

Figure S8. Total ion count chromatogram (TIC+) and mass spectral data for compound **PEDV 4** after cleavage of the assembled peptide from SPPS resin and HPLC purification. The solvent gradient (%B = acetonitrile) is highlighted in pink in the DAD chromatogram at 214nm.

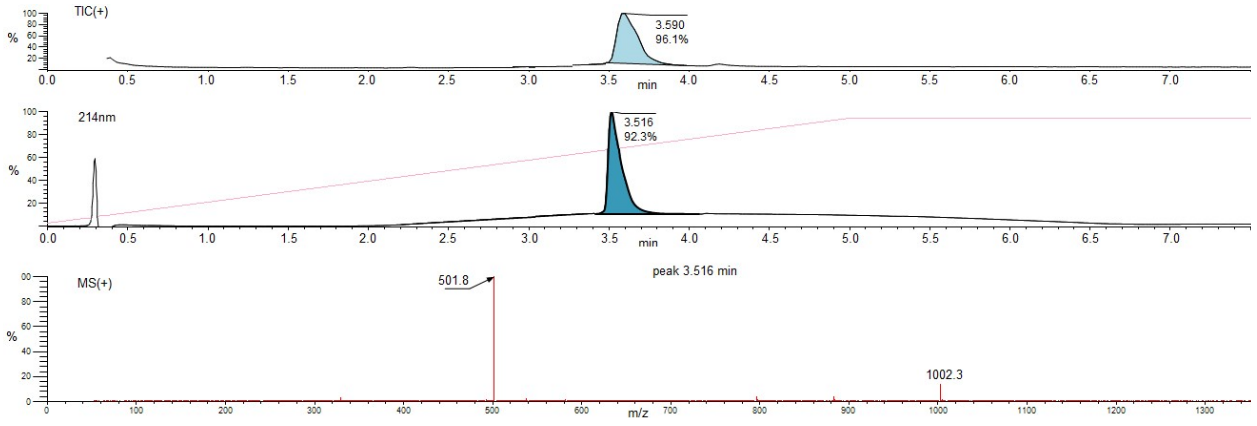

Figure S9. Total ion count chromatogram (TIC+) and mass spectral data for compound **(-)FRET** (negative control) after cleavage of the assembled peptide from SPPS resin and HPLC purification. The solvent gradient (%B = acetonitrile) is highlighted in pink in the DAD chromatogram at 214nm.

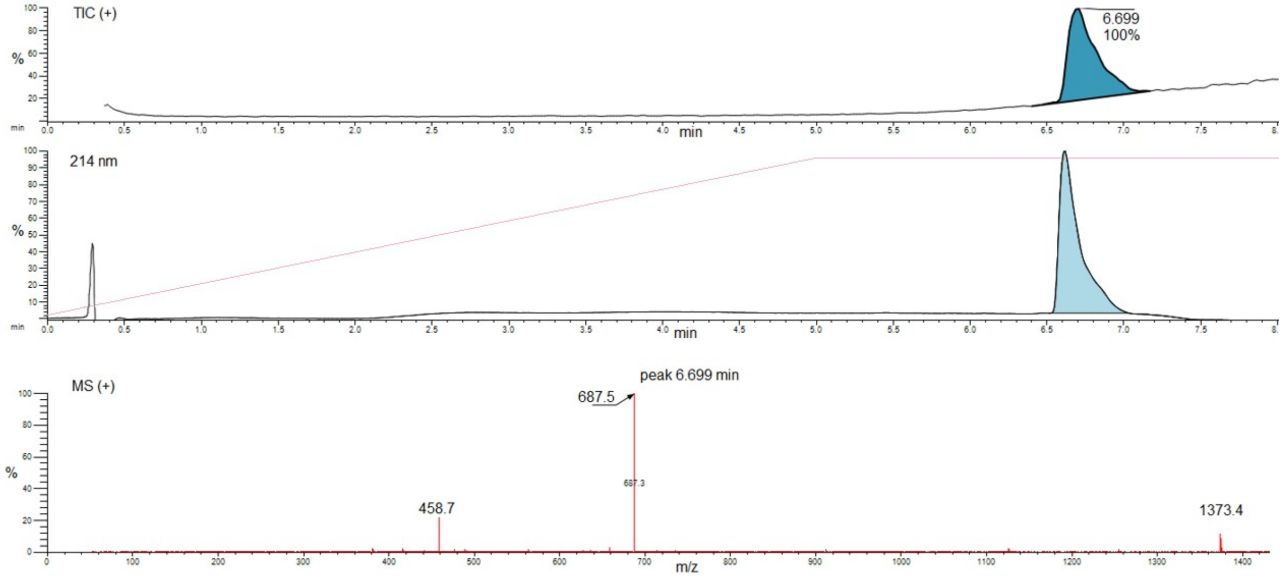

Figure S10. Total ion count chromatogram (TIC+) and mass spectral data for compound **HRVFRET** after cleavage of the assembled peptide from SPPS resin and HPLC purification. The solvent gradient (%B = acetonitrile) is highlighted in pink in the DAD chromatogram at 214nm.

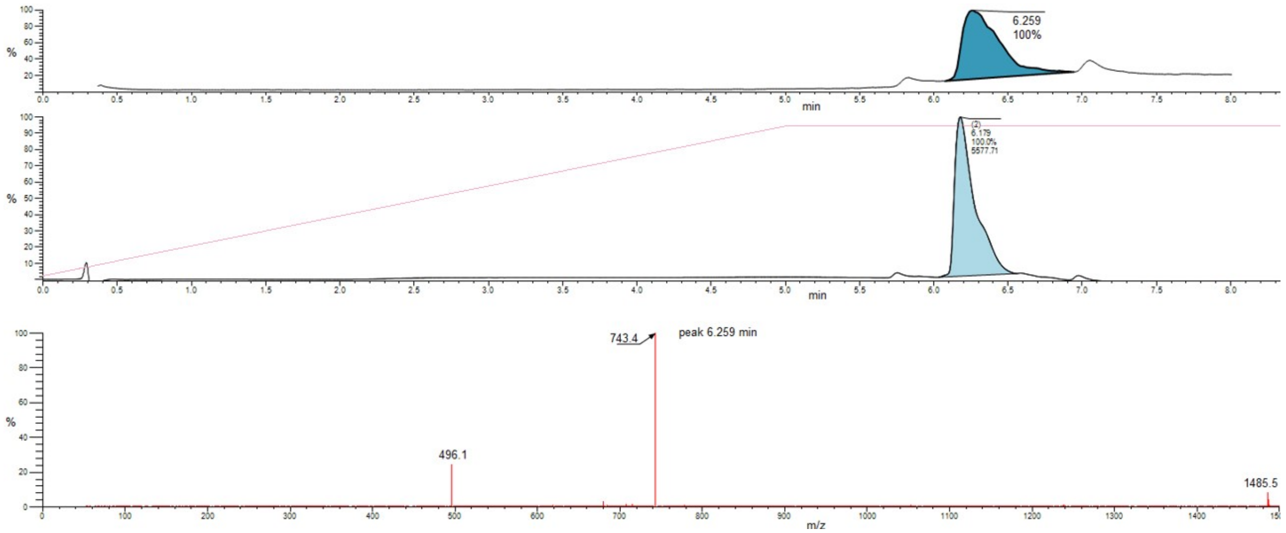

Figure S11. Total ion count chromatogram (TIC+) and mass spectral data for compound **PVFRET** after cleavage of the assembled peptide from SPPS resin and HPLC purification. The solvent gradient (%B = acetonitrile) is highlighted in pink in the DAD chromatogram at 214nm.

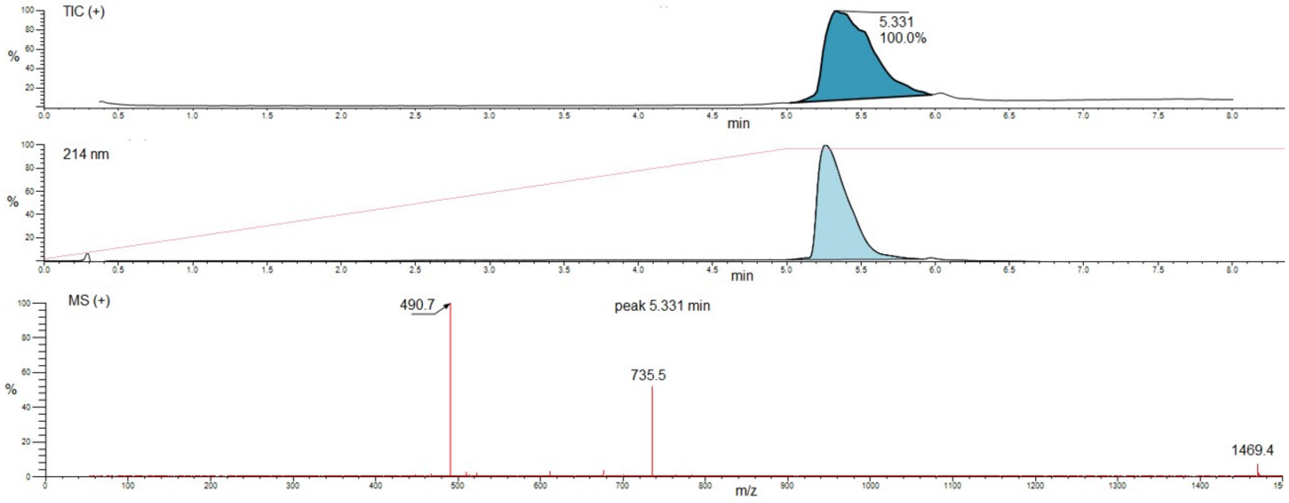

Figure S12. Total ion count chromatogram (TIC+) and mass spectral data for compound **EVFRET** after cleavage of the assembled peptide from SPPS resin and HPLC purification. The solvent gradient (%B = acetonitrile) is highlighted in pink in the DAD chromatogram at 214nm.

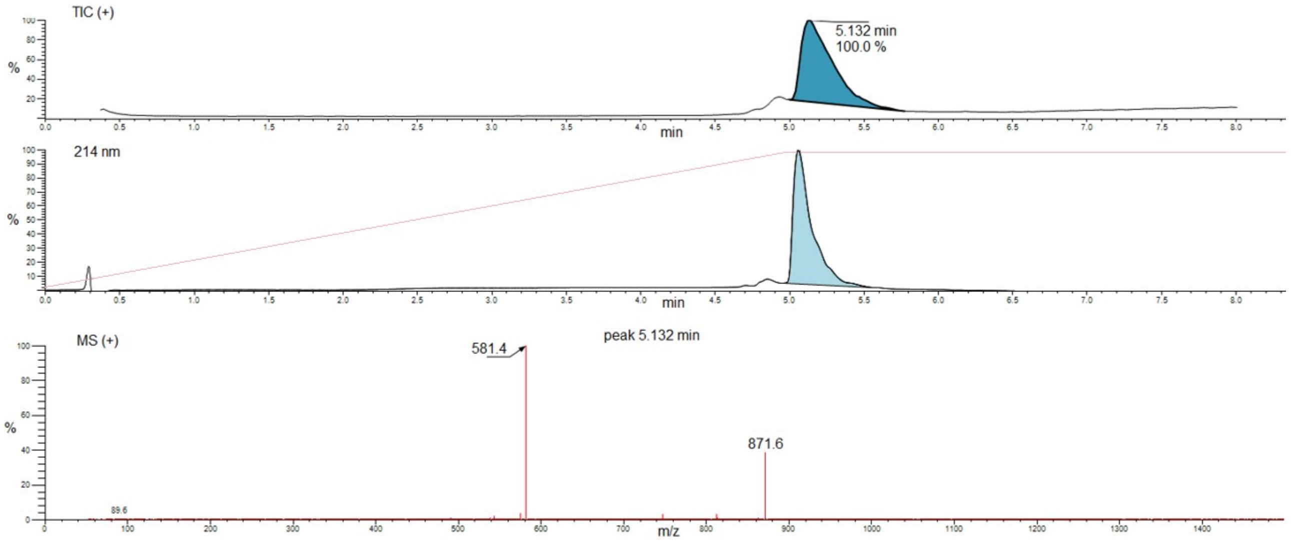

Figure S13. Total ion count chromatogram (TIC+) and mass spectral data for compound **NVFRET** after cleavage of the assembled peptide from SPPS resin and HPLC purification. The solvent gradient (%B = acetonitrile) is highlighted in pink in the DAD chromatogram at 214nm.

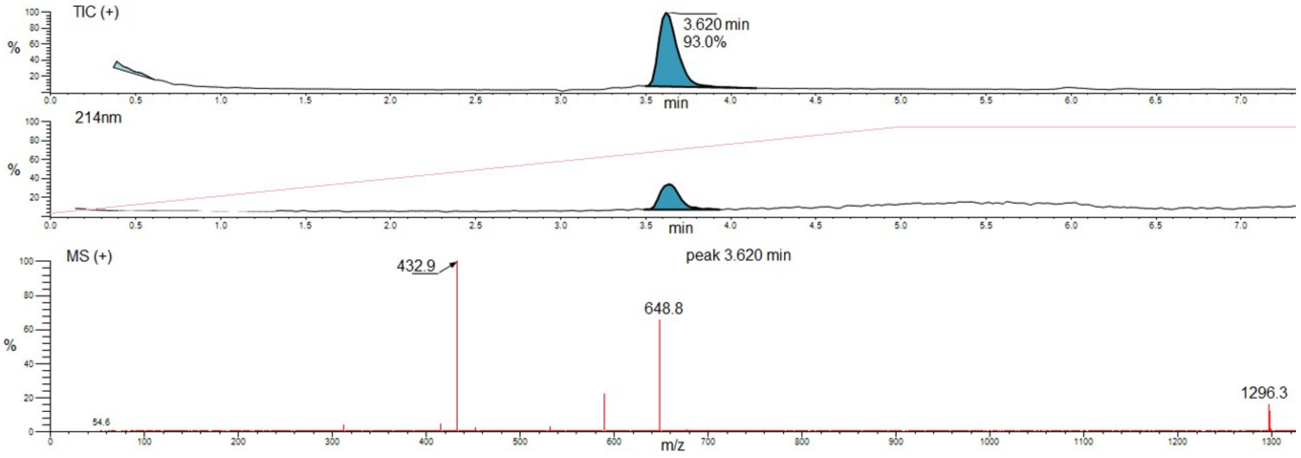

Figure S14. Total ion count chromatogram (TIC+) and mass spectral data for compound **NPFRET** after cleavage of the assembled peptide from SPPS resin and HPLC purification. The solvent gradient (%B = acetonitrile) is highlighted in pink in the DAD chromatogram at 214nm.

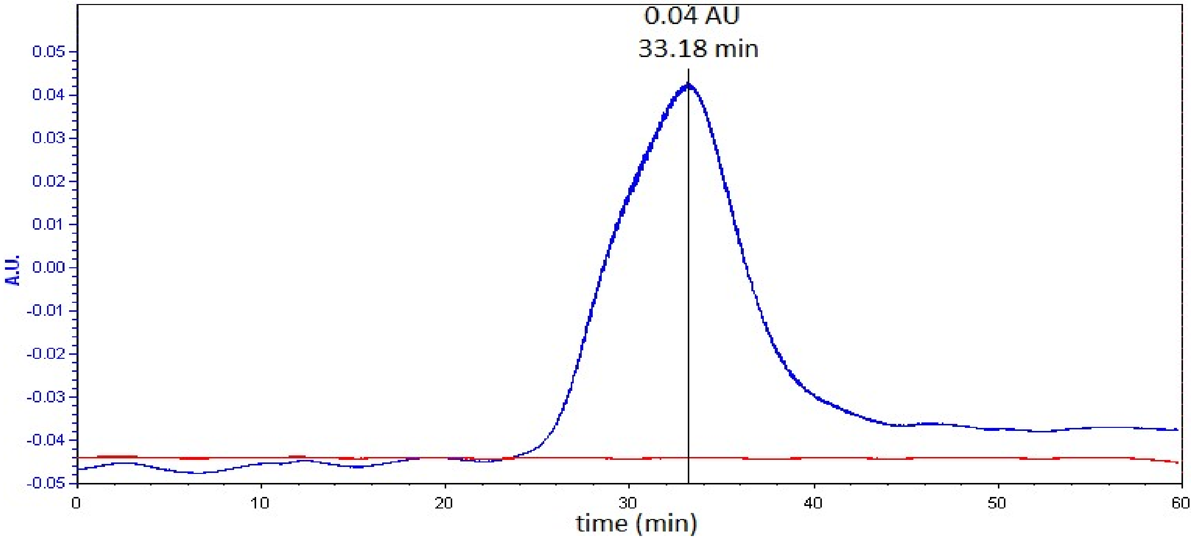

Gel filtration chromatogram (Sephadex G-15, 1 mL/min, 20 mM Bis-Tris, 150 mM NaCl, 1 mM DTT, 1 mM EDTA, pH 7.8, 0.02% Tween) of FIPV-M^{pro}

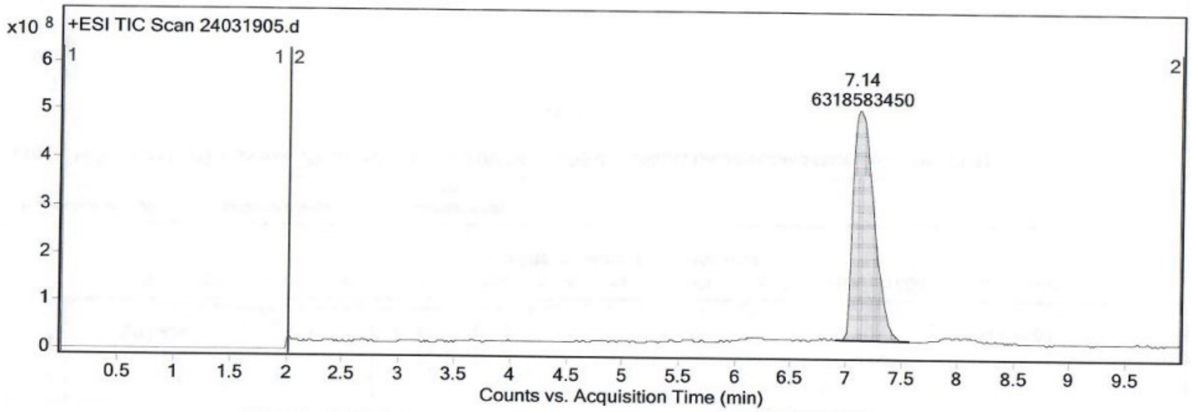

Integration Peak List

Peak	Start	RT	End	Height	Area	Area %
1	6.91	7.14	7.58	479186990	6318583450	100

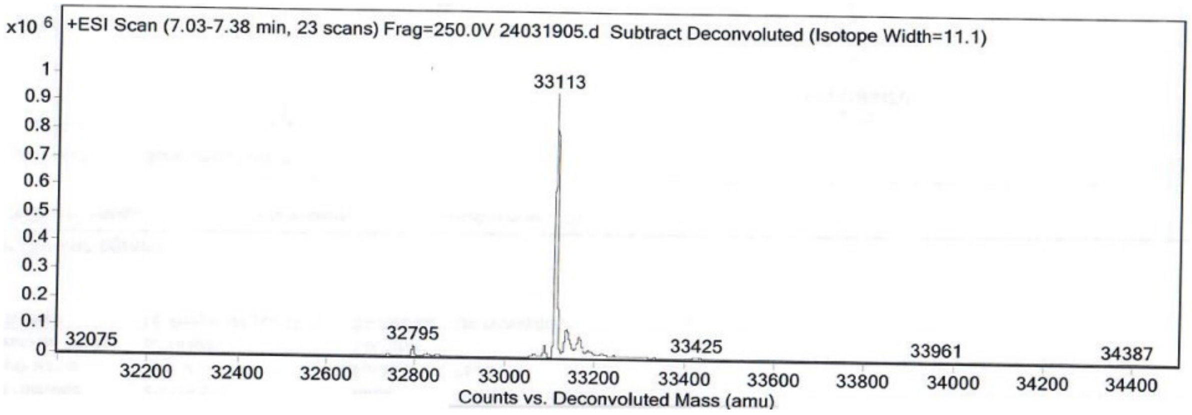

Chromatogram and deconvoluted mass spectrum of FIPV-M^{pro}

Figure S15. Chromatographic and ESI mass-spectrometric characterization of FIPV-M^{pro}.

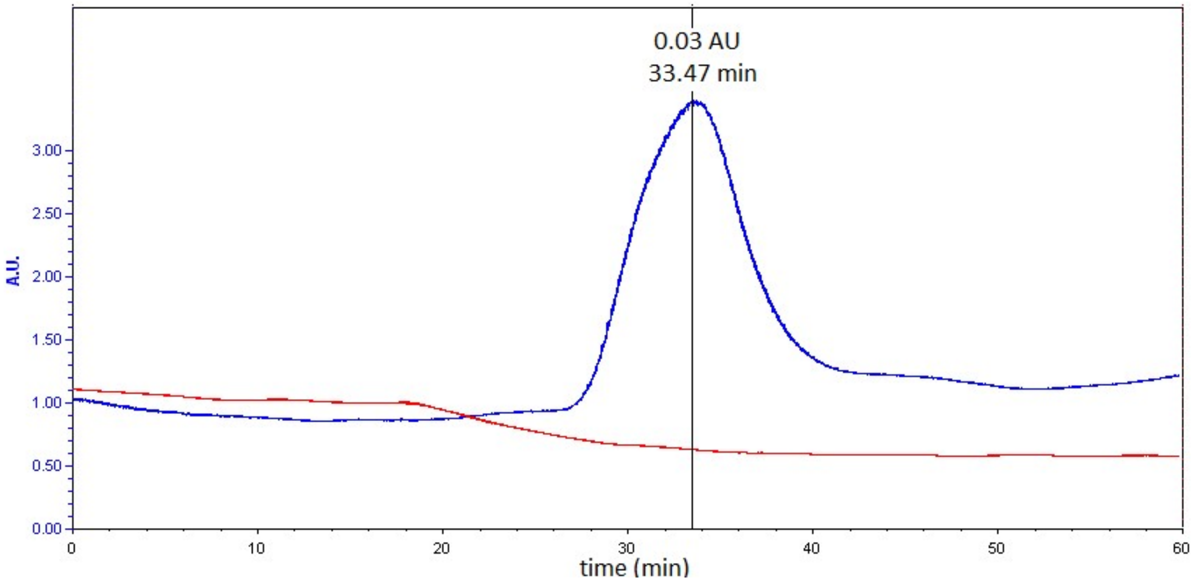

Gel filtration chromatogram (Sephadex G-15, 1 mL/min, 20 mM Bis-Tris, 150 mM NaCl, 1 mM DTT, 1 mM EDTA, pH 7.8, 0.02% Tween) of PEDV-M^{pro}.

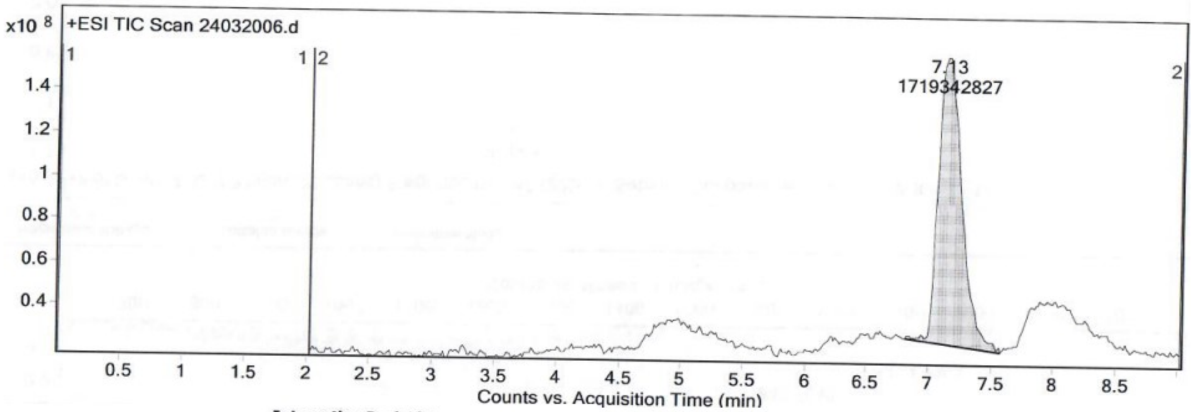

Integration Peak List

Peak	Start	RT	End	Height	Area	Area %
1	6.81	7.13	7.58	133078482	1719342827	100

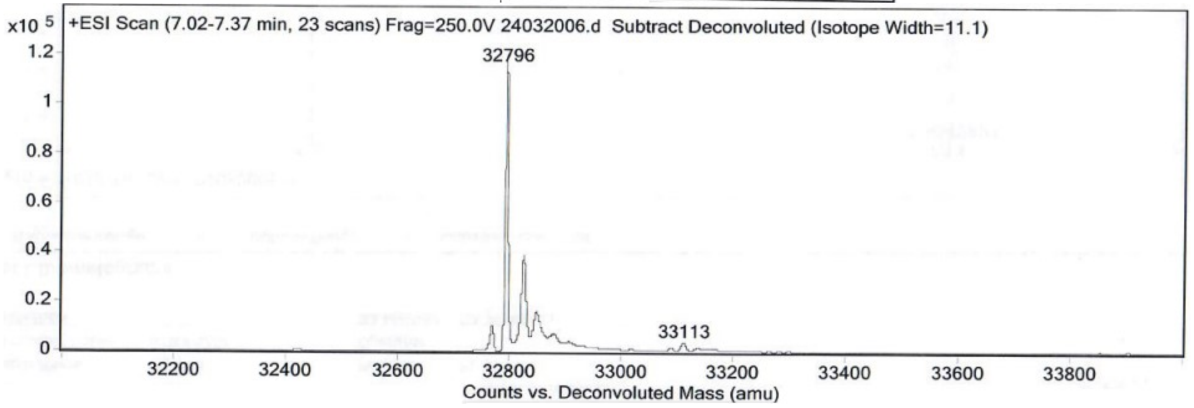

Chromatogram and deconvoluted mass spectrum of PEDV-M^{pro}.

Figure S16. Chromatographic and ESI mass-spectrometric characterization of PEDV-M^{pro}.

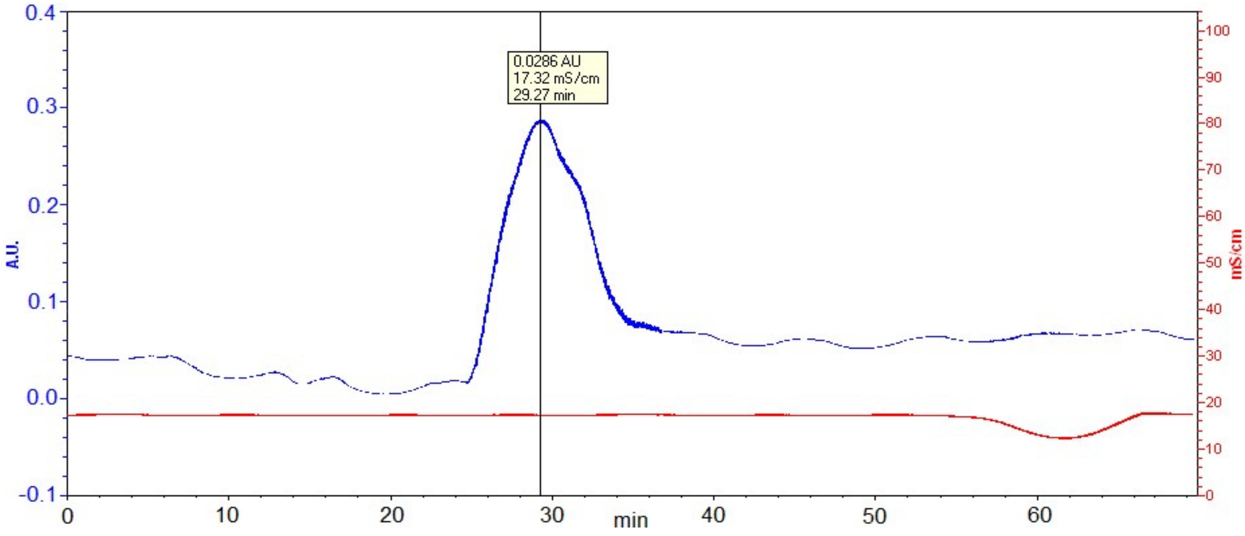

Gel filtration chromatogram (Sephadex G-15, 1 mL/min, 20 mM Bis-Tris, 150 mM NaCl, 1 mM DTT, 1 mM EDTA, pH 7.8, 0.02% Tween) of EqCoV-M^{pro}.

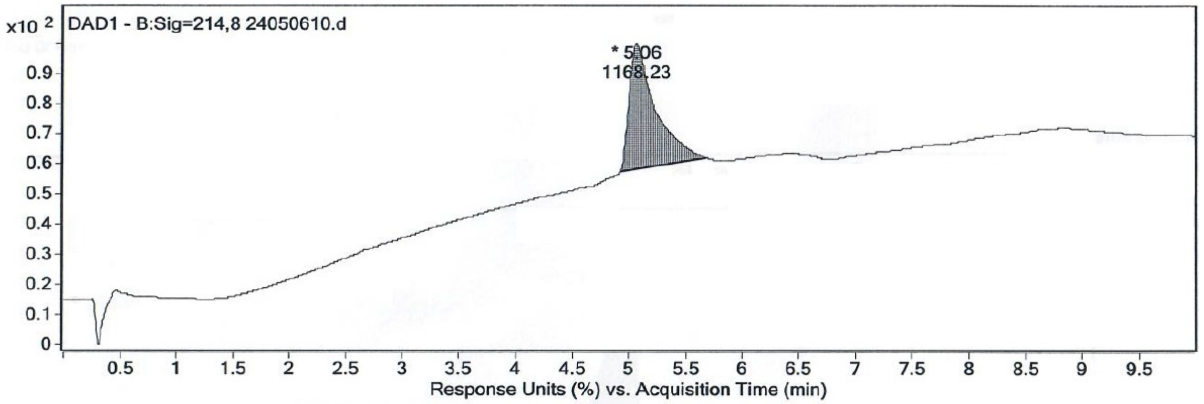

Integration Peak List

Peak	Start	RT	End	Height	Area	Area %
1	4.92	5.06	5.67	73.57	1168.23	100

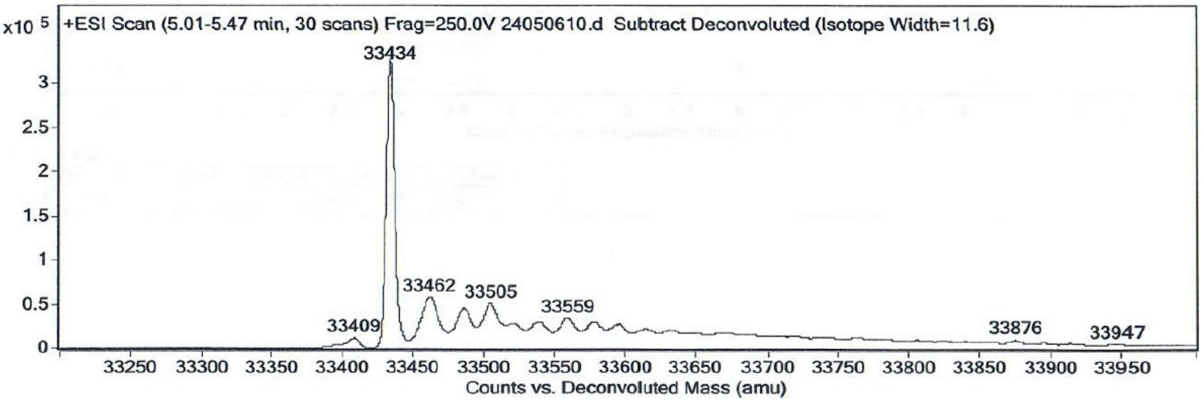

Chromatogram and deconvoluted mass spectrum of EqCoV-M^{pro}.

Figure S17. Chromatographic and ESI mass-spectrometric characterization of EqCoV-M^{pro}.

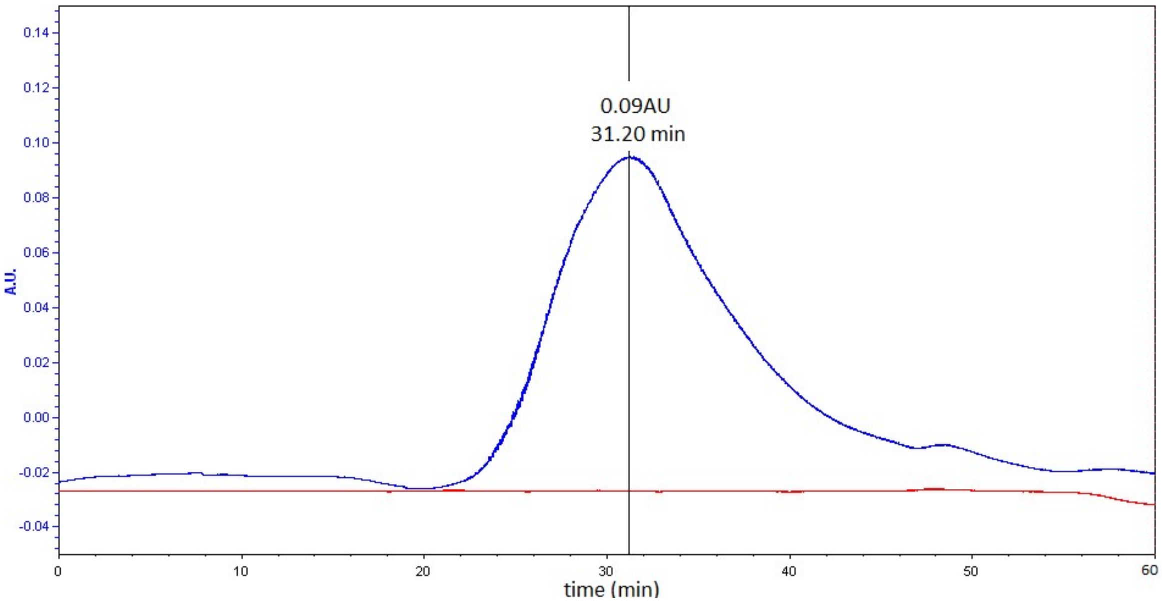

Gel filtration chromatogram (Sephadex G-15, 1 mL/min, 20 mM Bis-Tris, 150 mM NaCl, 1 mM DTT, 1 mM EDTA, pH 7.8, 0.02% Tween) of HRV-M^{pro}

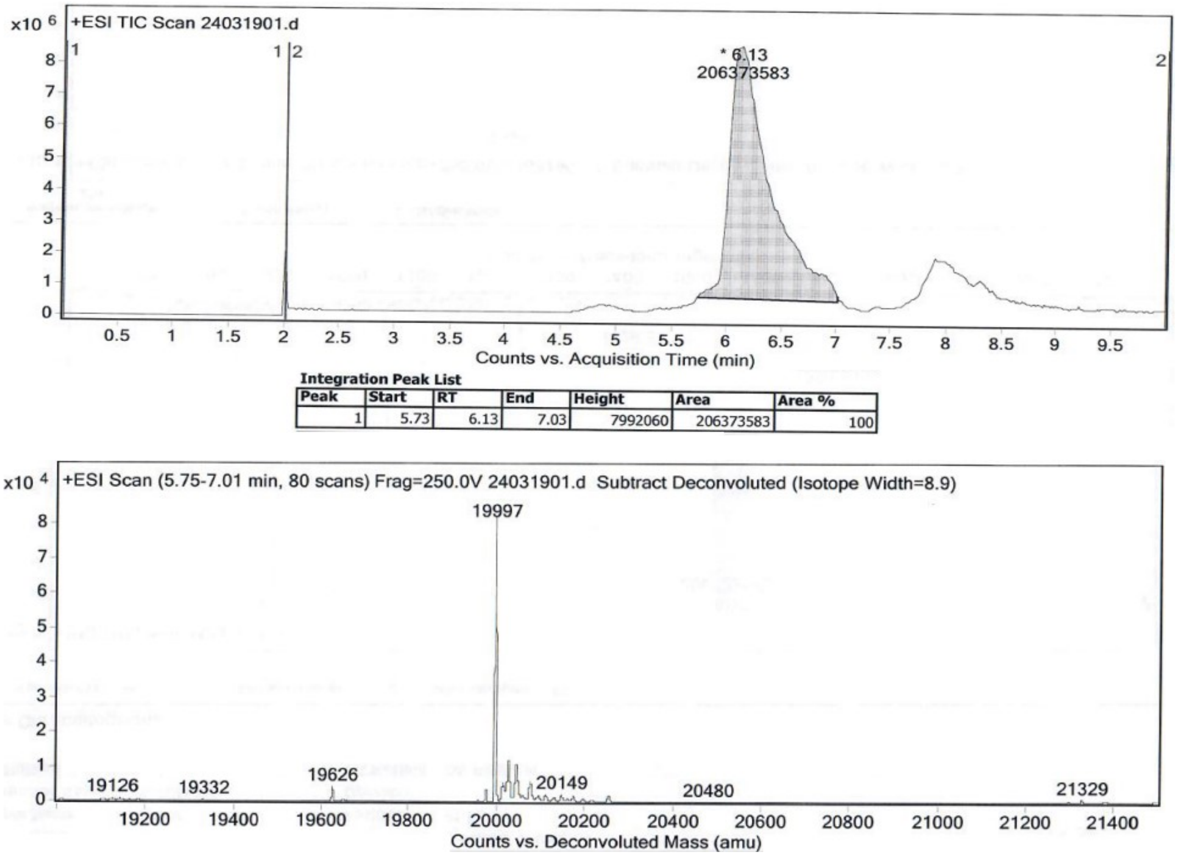

Chromatogram and deconvoluted mass spectrum of HRV-M^{pro}

Figure S18. Chromatographic and ESI mass-spectrometric characterization of HRV-M^{pro}.

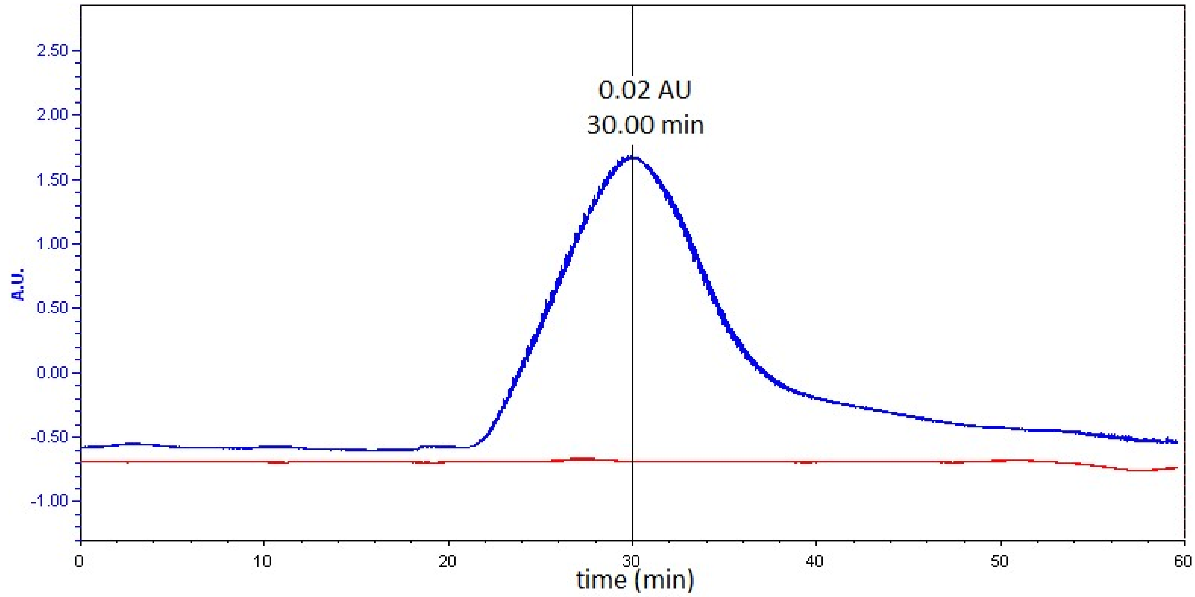

Gel filtration chromatogram (Sephadex G-15, 1 mL/min, 20 mM Bis-Tris, 150 mM NaCl, 1 mM DTT, 1 mM EDTA, pH 7.8, 0.02% Tween) of PV-M^{pro}

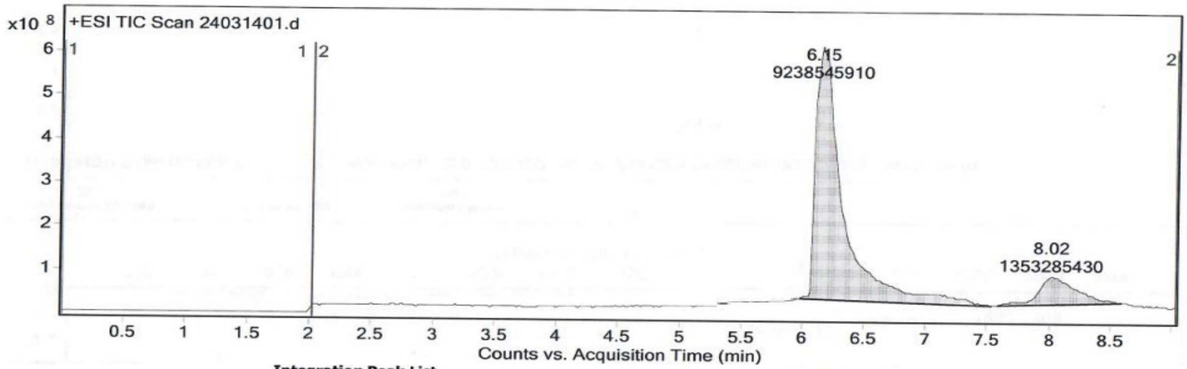

Integration Peak List

Peak	Start	RT	End	Height	Area	Area %
1	5.91	6.15	7.54	578479910	9238545910	100
2	7.59	8.02	8.59	62173210	1353285430	14.65

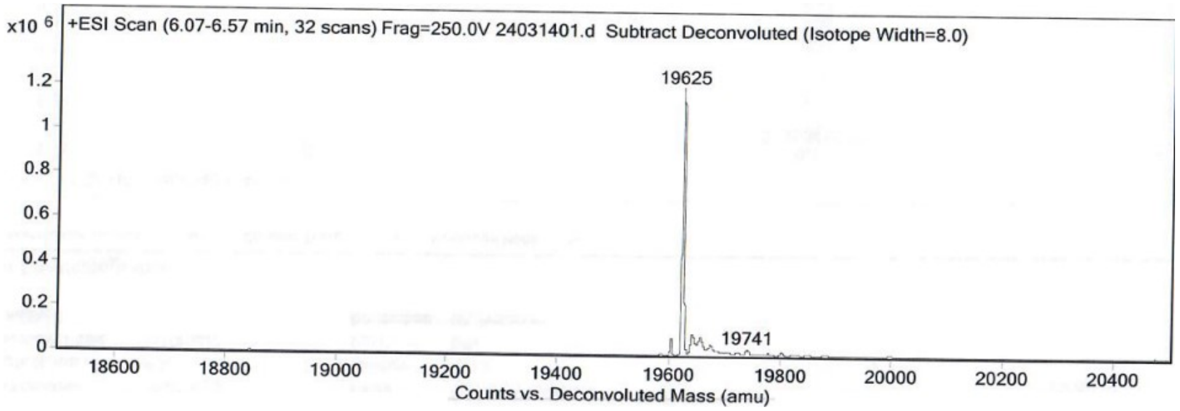

Chromatogram and deconvoluted mass spectrum of PV-M^{pro}

Figure S19. Chromatographic and ESI mass-spectrometric characterization of PV-M^{pro}.

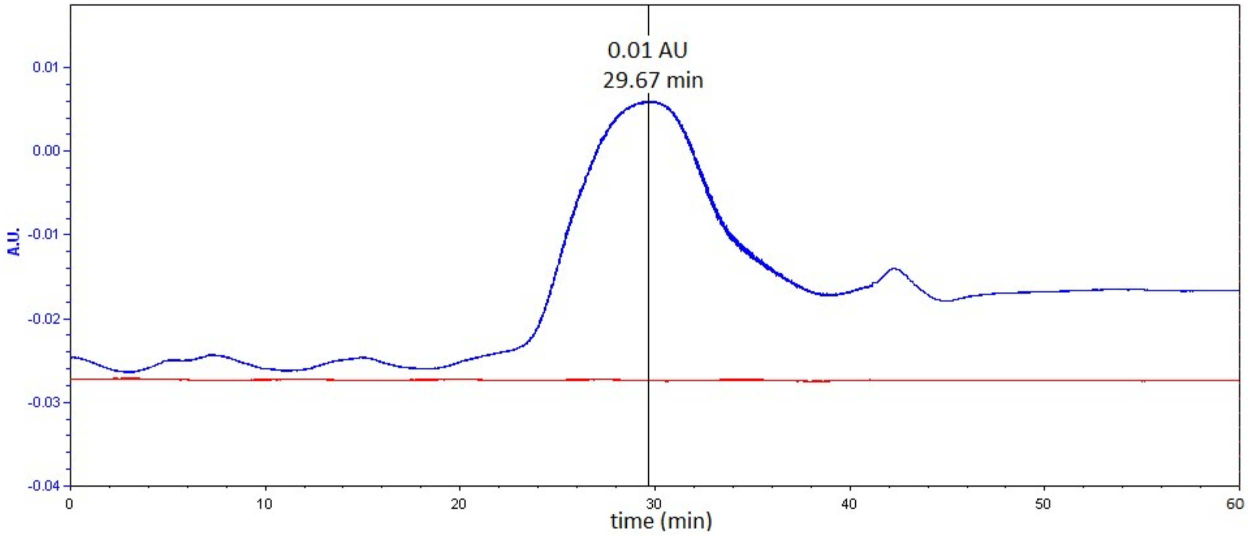

Gel filtration chromatogram (Sephadex G-15, 1 mL/min, 20 mM Bis-Tris, 150 mM NaCl, 1 mM DTT, 1 mM EDTA, pH 7.8, 0.02% Tween) of EV71-M^{pro}

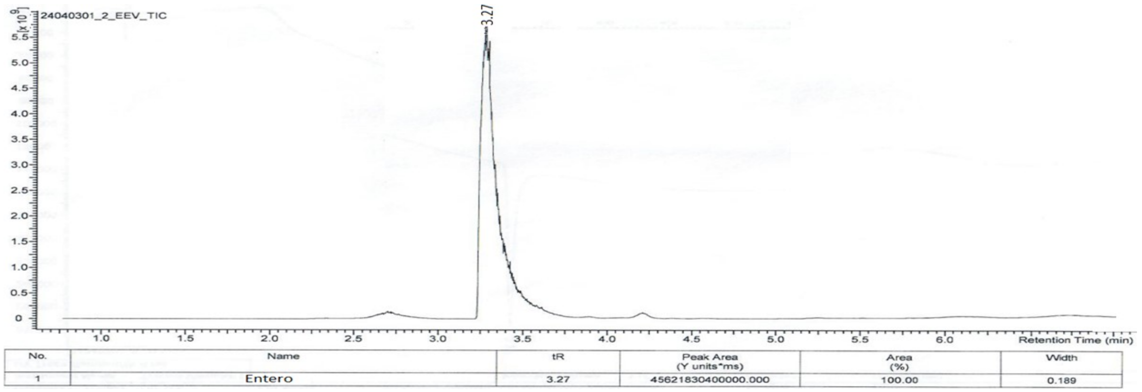

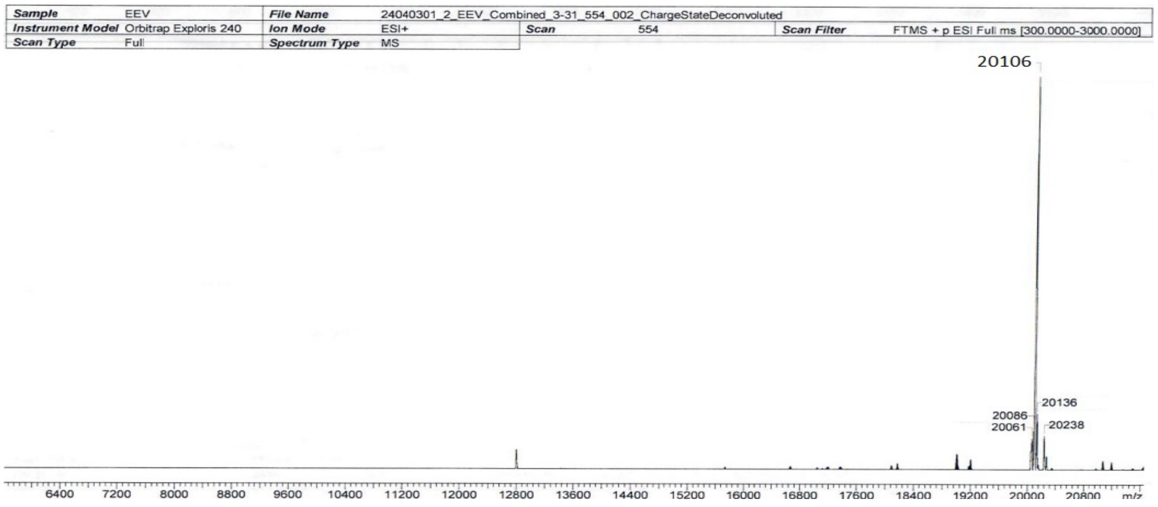

Chromatogram and deconvoluted mass spectrum of EV71-M^{pro}

Figure S20. Chromatographic and ESI mass-spectrometric characterization of EV71-M^{pro}.

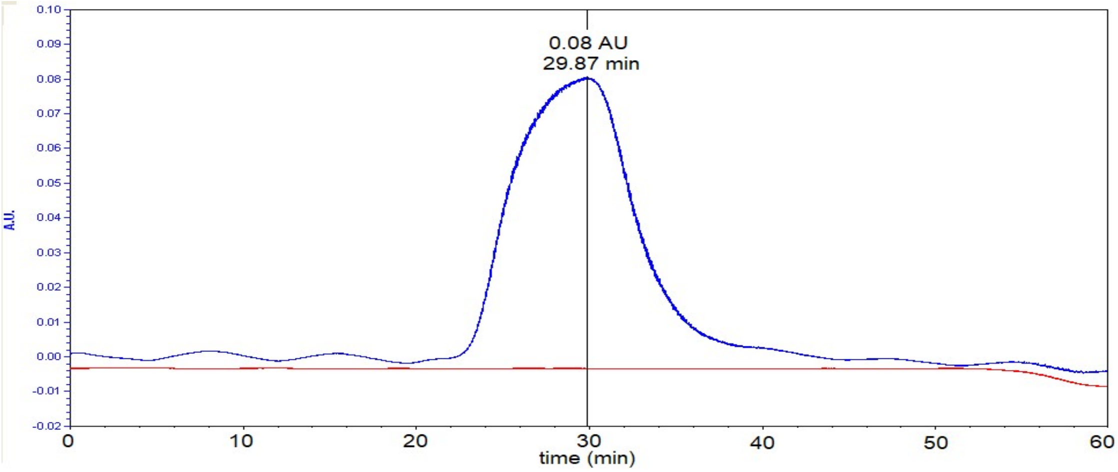

Gel filtration chromatogram (Sephadex G-15, 1 mL/min, 20 mM Bis-Tris, 150 mM NaCl, 1 mM DTT, 1 mM EDTA, pH 7.8, 0.02% Tween) of NV-M^{pro}

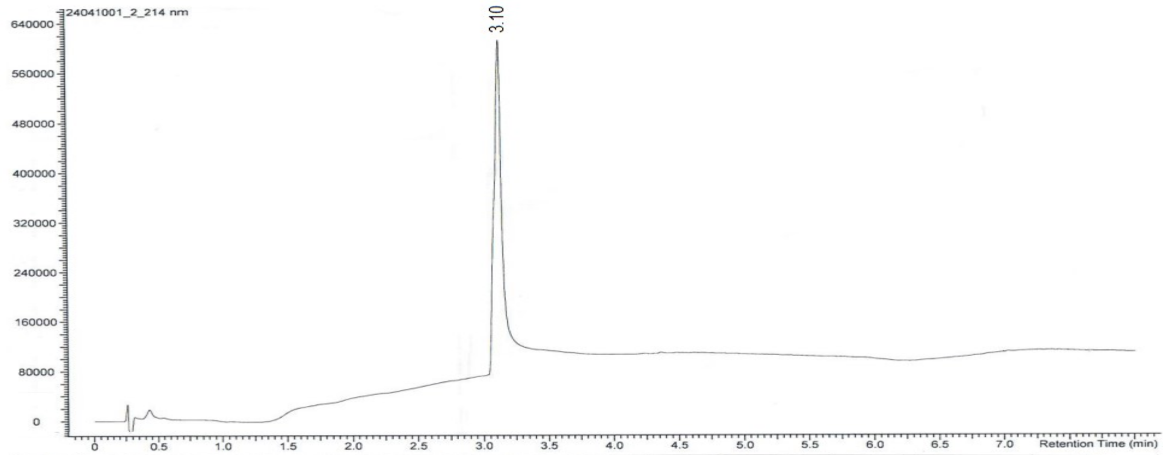

No.	Name	tR	Peak Area (Y units*ms)	Area (%)	Width
1	NV	3.10	3305186953.125	100.00	0.097

Sample	NV	*File Name*	24041001_1_NV-36_Combined_3-65_574_003_ChargeStateDeconvoluted				
Instrument Model	Orbitrap Exploris 240	*Ion Mode*	ESI+	*Scan*	574	*Scan Filter*	FTMS + p ESI Full ms [300.0000-3000.0000]
Scan Type	Full	*Spectrum Type*	MS				

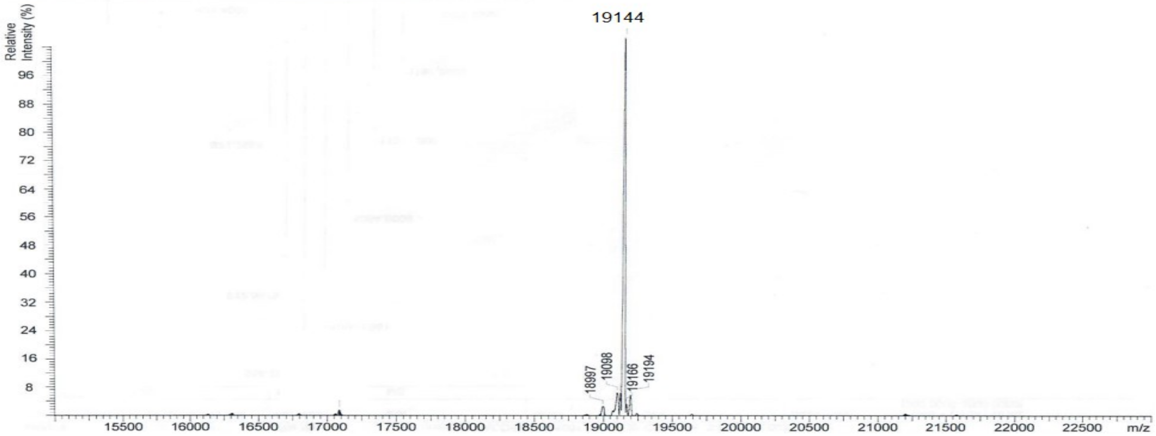

Chromatogram and deconvoluted mass spectrum of NV-M^{pro}

Figure S21. Chromatographic and ESI mass-spectrometric characterization of NV-M^{pro}.

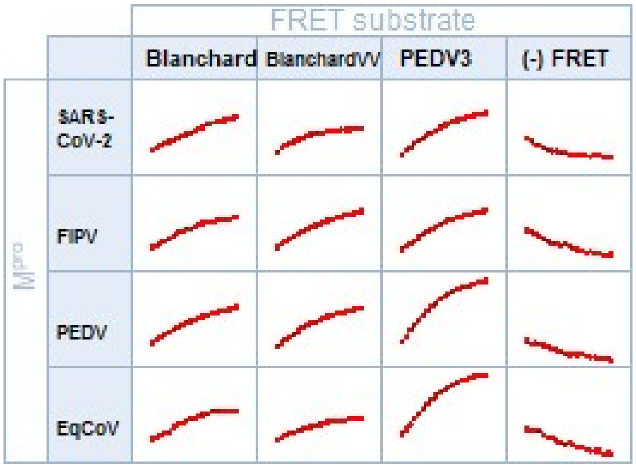

Figure S22. Reaction progression curves of various M^pro^s with selected FRET substrates. The curves depict fluorescence released over the course of a 10 min assay.

Blanchard: 2-AbzSVTLQ↓SGY$^{(NO2)}$R —SARS-CoV-2 M^{pro}→ **2-AbzSVTLQ** + **SGY$^{(NO2)}$R**

1174 g/mol 666 g/mol 526 g/mol

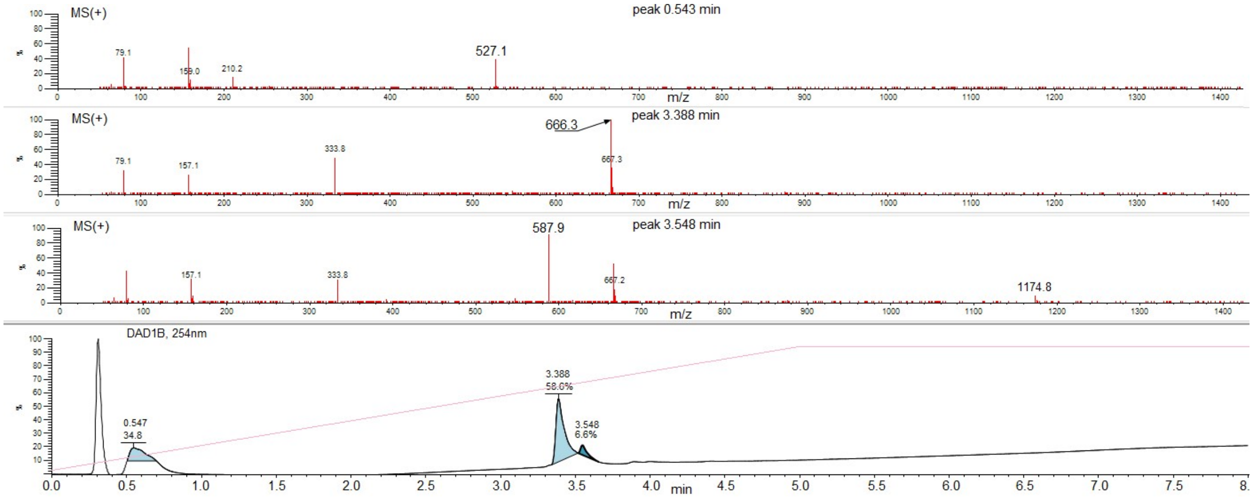

PEDV3: 2-AbzSTLQ↓SGY$^{(NO2)}$R —SARS-CoV-2 M^{pro}⟶ **2-AbzSTLQ** + **SGY$^{(NO2)}$R**

1075 g/mol 566 g/mol 526 g/mol

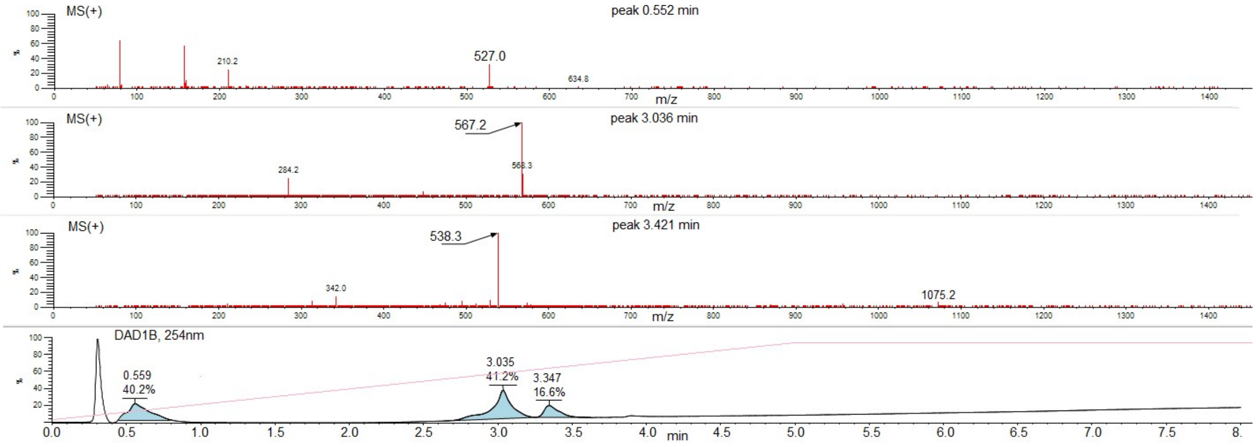

(-) FRET: 2-AbzSTLAAGY$^{(NO2)}$R —SARS-CoV-2 M^{pro}⟶ no cleavage

1002 g/mol

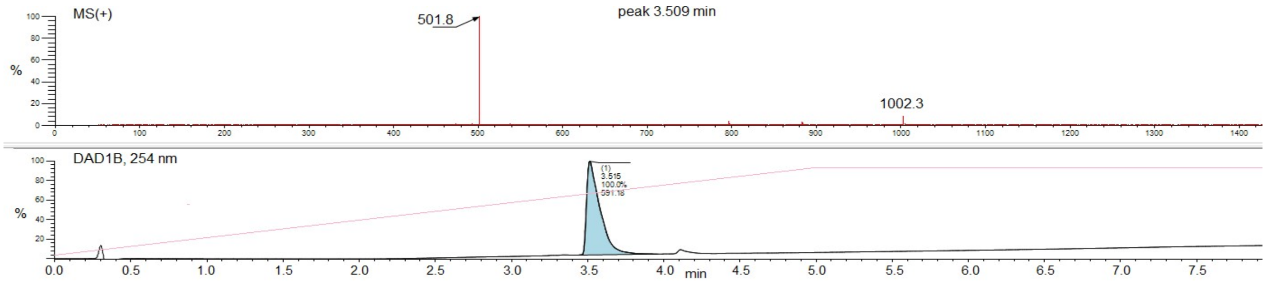

Figure S23. LCMS analysis of cleavage products formed by reaction of Blanchard FRET (top) and PEDV3 FRET (middle) and negative control ((-)FRET, bottom) with SARS-CoV-2 M^{pro} after 10 min cleavage highlighting cleavage specificity of the main protease.

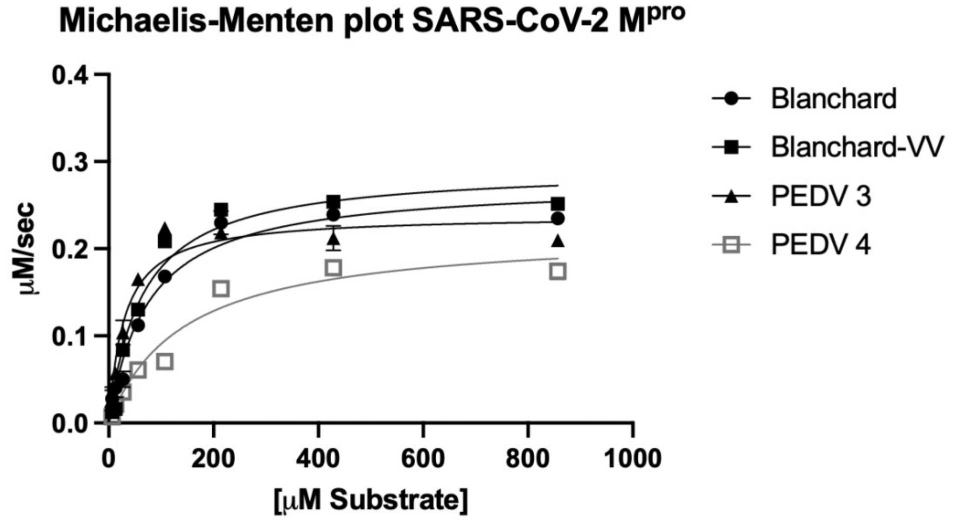

	Blanchard	Blanchard-VV	PEDV 3	PEDV 4
v_{max}, µM s^{-1}	0.2763±0.02	0.2927±0.02	0.2386±0.04	0.2201±0.03
K_M, µM	82±14	64±10	30±8	142±18
Goodness of fit				
R squared	0.9664	0.9619	0.9321	0.9545

Figure S24. Michaelis-Menten plot of various coronavirus FRETs with SARS-CoV-2 M^{pro} .

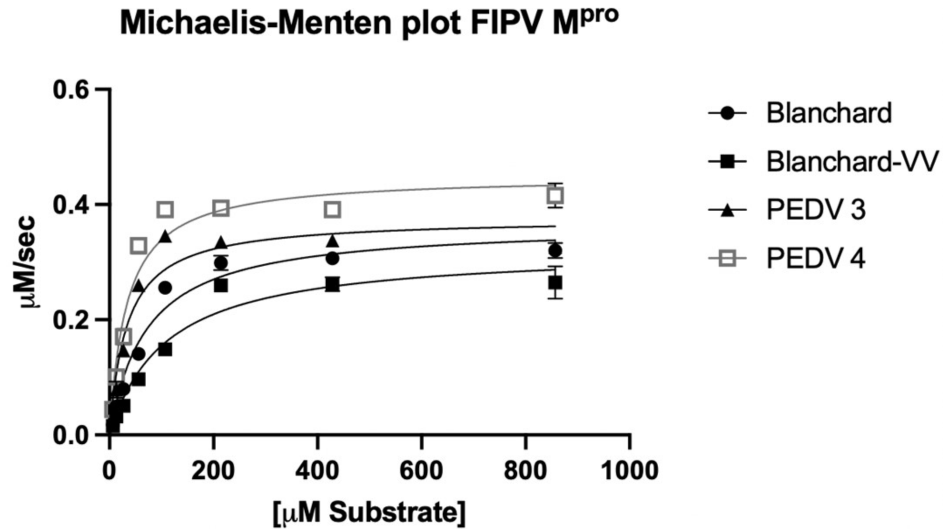

	Blanchard	Blanchard-VV	PEDV 3	PEDV 4
v_{max}, µM s^{-1}	0.3661±0.03	0.3231±0.04	0.3762±0.04	0.4199±0.04
K_M, µM	72±12	85±15	33.1±12	32.9±11
Goodness of fit				
R squared	0.9675	0.9556	0.9322	0.9471

Figure S25. Michaelis-Menten plot of various coronavirus FRETs with FIPV M^{pro} .

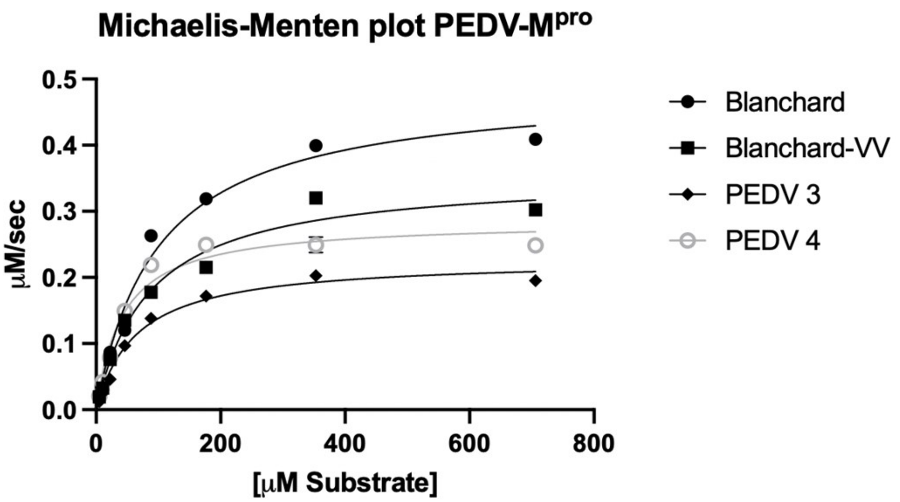

	Blanchard	Blanchard-VV	PEDV 3	PEDV 4
v_{max}, μM s^{-1}	0.489±0.03	0.360±0.04	0.269±0.03	0.228±0.01
K_M, μM	100±18	85±10	38±11	65±12
Goodness of fit				
R squared	0.9817	0.9801	0.9713	0.9892

Figure S26. Michaelis-Menten plot of various coronavirus FRETs with PEDV M^{pro} .

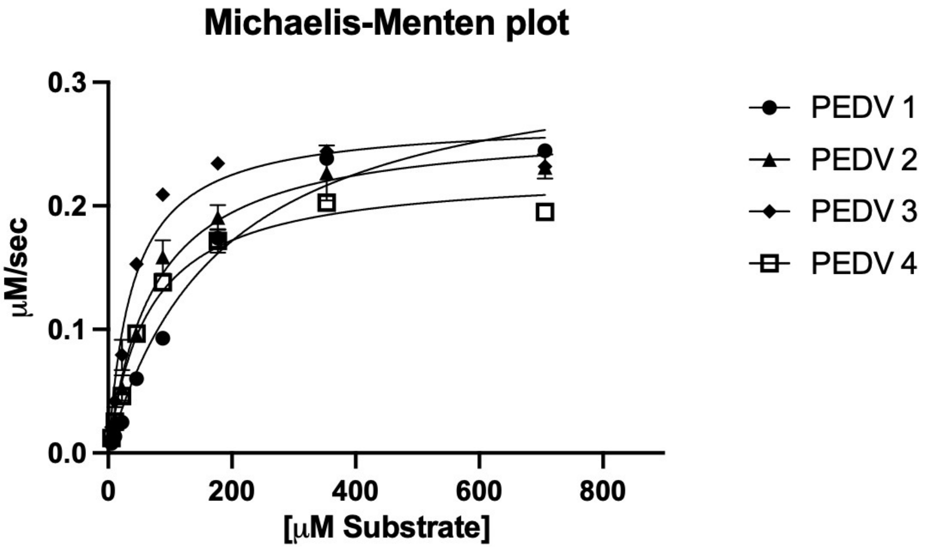

	PEDV 1	PEDV 2	PEDV 3	PEDV 4
v_{max}, μM s^{-1}	0.330±0.02	0.266±0.02	0.269±0.03	0.248±0.01
K_M, μM	192±26	63±11	38±11	65±12
Goodness of fit				
R squared	0.9852	0.9819	0.9713	0.9892

Figure S27. Michaelis-Menten plot of various PEDV FRETs with PEDV M^{pro}.

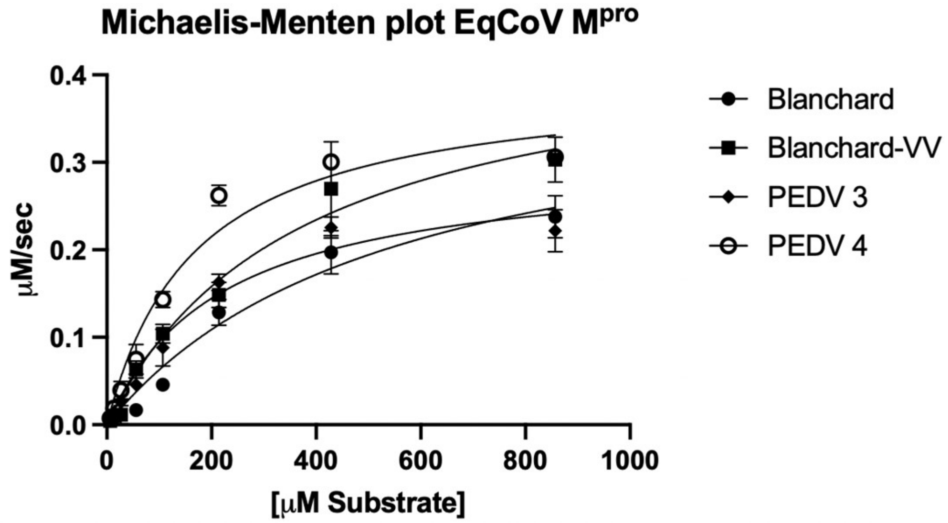

	PEDV 1	PEDV 2	PEDV 3	PEDV 4
v_{max}, μM s^{-1}	0.4075	0.4516	0.3027	0.3963
K_M, μM	549.1	371.1	219.1	168.5
Goodness of fit				
R squared	0.9632	0.9630	0.9623	0.9664

Figure S28. Michaelis-Menten plot of various coronavirus FRETs with EqCoV M^{pro} .

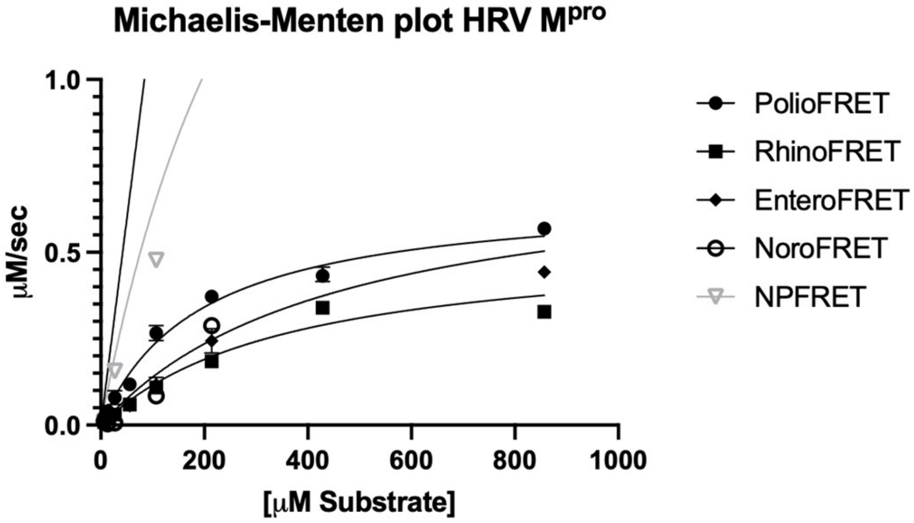

	PVFRET	HRVFRET	EVFRET	NVFRET	NPFRET
v_{max}, μM s^{-1}	0.6694±0.06	0.5348±0.08	0.7709±0.08	n.d.*	n.d.*
K_M, μM	192.1±35	363.0±83	455.3±112	n.d.*	n.d.*
Goodness of fit					
R squared	0.9836	0.9593	0.9554	0.8414	0.07005

Figure S29. Michaelis-Menten plot of various picornavirus FRETs with HRV M^{pro} . No exact data for entries with (*) could be determined due lack of convergence.

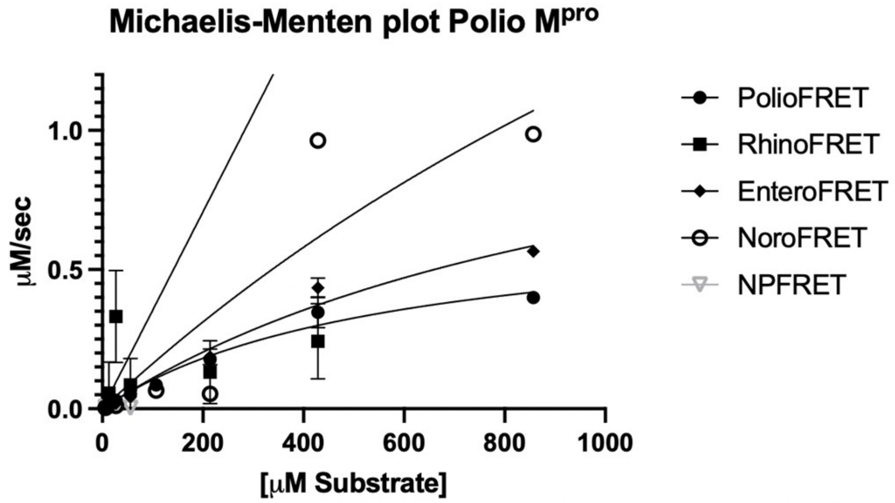

	PVFRET	HRVFRET	EVFRET	NVFRET	NPFRET
v_{max}, μM s^{-1}	0.6969±0.06	n.d.*	1.366±0.30	4.136±1.36	n.d.*
K_M, μM	569.3±95	n.d.*	1143±205	2452±830	1386
Goodness of fit					
R squared	0.9693	0.7278	0.9756	0.8315	0.7265

Figure S30. Michaelis-Menten plot of various picornavirus FRETs with PV M^{pro} . No exact data for entries with (*) could be determined due lack of convergence.

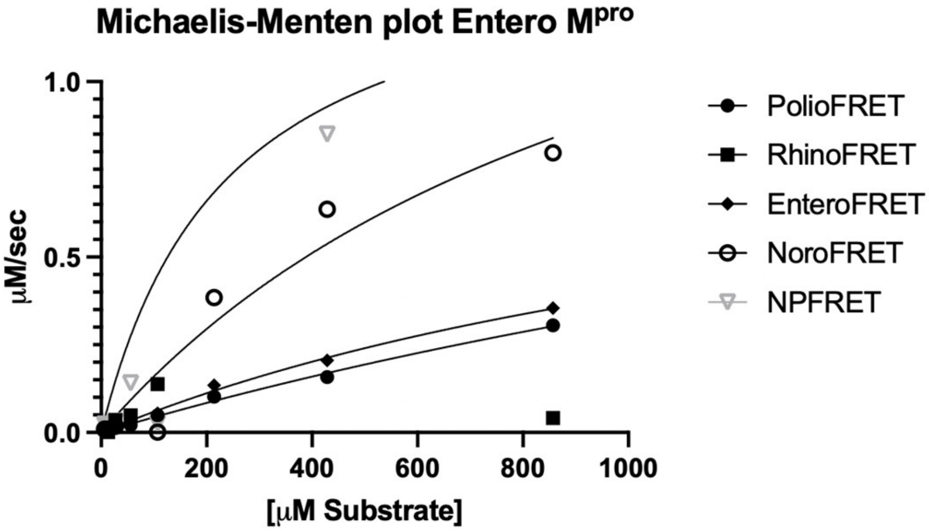

	PVFRET	HRVFRET	EVFRET	NVFRET	NPFRET
v_{max}, μM s^{-1}	1.442±0.21	n.d.*	1.029±0.13	1.914±0.29	n.d.*
K_M, μM	3223	237.1	1641	1097	n.d.*
Goodness of fit					
R squared	0.9956	0.2027	0.9957	0.9246	0.04465

Figure S31. Michaelis-Menten plot of various picornavirus FRETs with EV71 M^{pro} . No exact data for entries with (*) could be determined due lack of convergence.

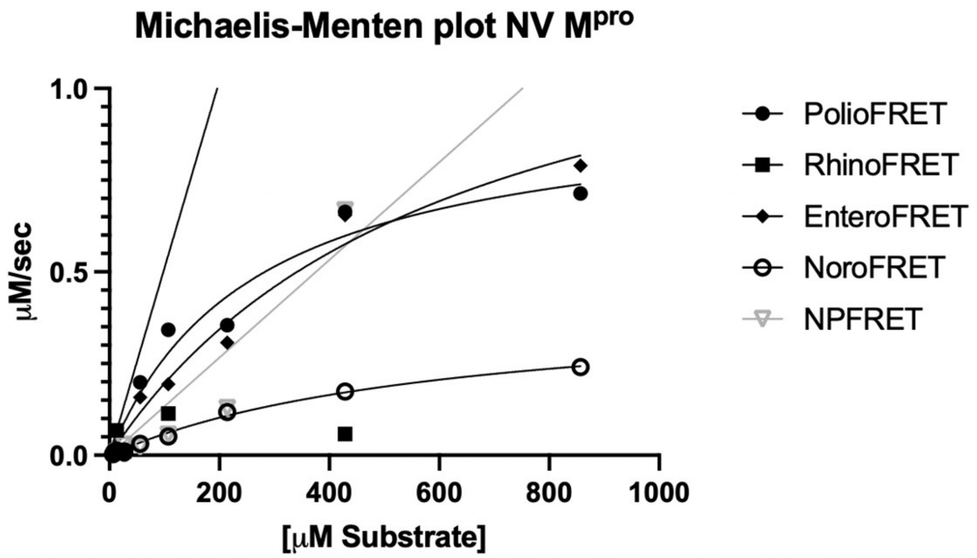

	PVFRET	HRVFRET	EVFRET	NVFRET	NPFRET
v_{max}, μM s^{-1}	0.9659±0.10	n.d.*	1.410±0.16	0.4163±0.08	n.d.*
K_M, μM	263.5	n.d.*	621.5	613.0	n.d.*
Goodness of fit					
R squared	0.9565	0.7016	0.9735	0.9945	0.9643

Figure S32. Michaelis-Menten plot of various picornavirus FRETs with NV M^{pro} . No exact data for entries with (*) could be determined due lack of convergence.

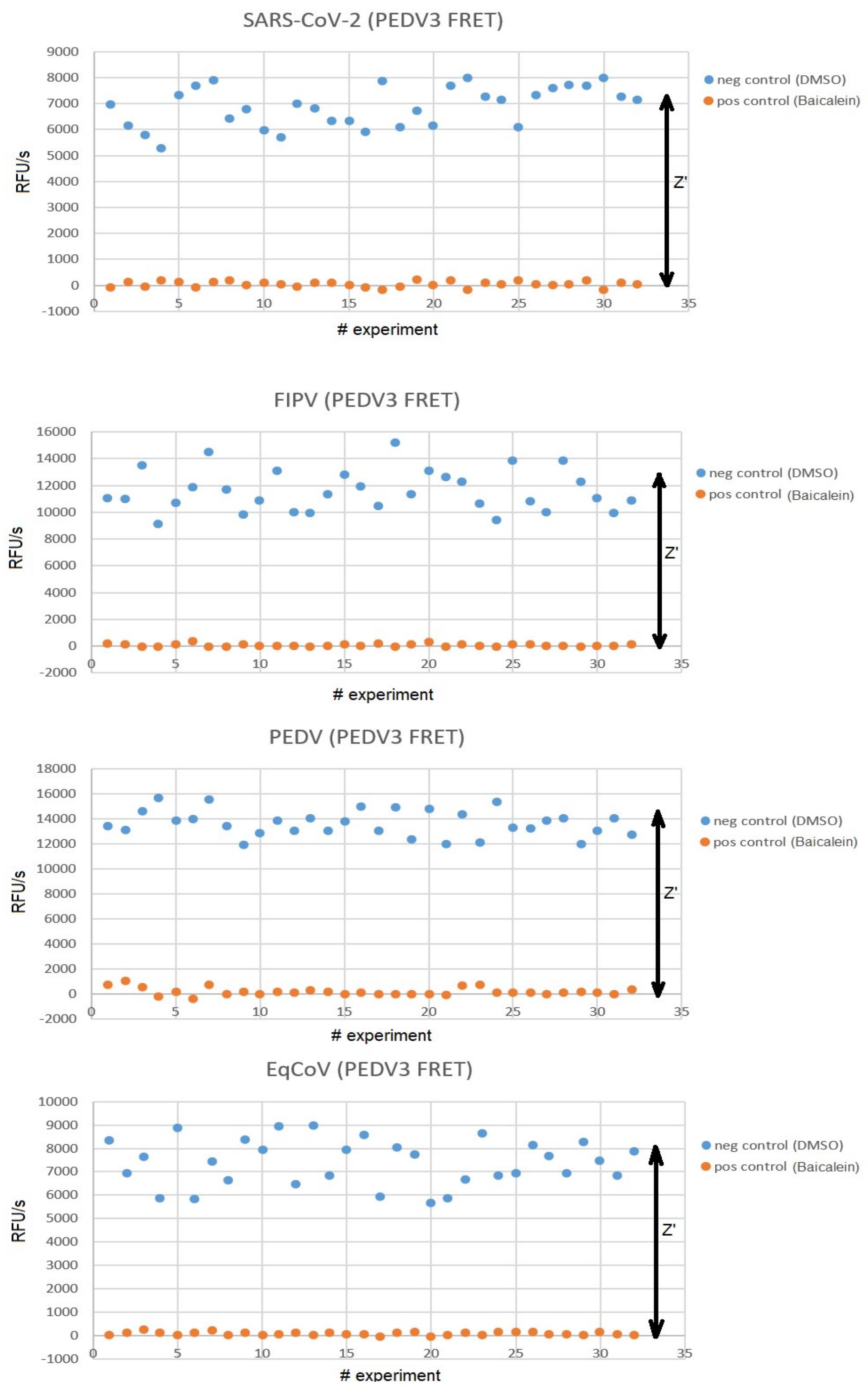

Figure S33. Z' assay quality statistics for different coronavirus M^{pro}s (100 nM) with PEDV 3 FRET substrate (10 µM). Samples were prepared with 50 µM baicalein (positive control) or 50 µM DMSO (negative control) and repeated in duplicate for *n*=32.
